# A summary of bird mortality at photovoltaic utility scale solar facilities in the Southwestern U.S.

**DOI:** 10.1371/journal.pone.0232034

**Published:** 2020-04-24

**Authors:** Karl Kosciuch, Daniel Riser-Espinoza, Michael Gerringer, Wallace Erickson

**Affiliations:** Western EcoSystems Technology, Inc., Cheyenne, Wyoming, United States of America; Stanford University, UNITED STATES

## Abstract

Recent trends in renewable energy development in the United States (U.S.) show that new installed capacity of utility-scale solar energy has exceeded 30% of total installed capacity of all sources per year since 2013. Photovoltaic solar energy provides benefits in that no emissions are produced; however, there are potential impacts from photovoltaic solar development on birds that include habitat loss and potential for collision mortality. Only 2 papers in the peer-reviewed literature present fatality information from fatality monitoring studies at a photovoltaic utility-scale solar energy facility; however, more data exists in unpublished reports. To provide a more comprehensive overview of bird mortality patterns, we synthesized results from fatality monitoring studies at 10 photovoltaic solar facilities across 13 site-years in California and Nevada. We found variability in the distribution of avian orders and species among and within Bird Conservation Regions, and found that water-obligate birds, which rely on water for take-off and landing, occurred at 90% (9/10) of site-years in the Sonoran and Mojave Deserts Bird Conservation Region. We found that a cause of mortality could not be determined for approximately 61% of intact carcasses, and that approximately 54% of all carcasses were feather spots, introducing uncertainty into the interpretation of the fatality estimates. The average annual fatality estimate we calculated for photovoltaic solar (high-end estimate of 2.49 birds per megawatt per year) is lower than that reported by another study (9.9 birds per megawatt per year) that included one photovoltaic facility. Our results provide a summary of fatalities in bird conservation regions where the facilities are located, but expanding our conclusions to new regions is limited by the location of facilities with fatality monitoring data.

## Introduction

Recent trends in renewable energy development in the United States (U.S.) show that new installed capacity of utility-scale solar energy (USSE) has exceeded 30% of total installed capacity of all energy sources per year since 2013 [1]. The development trend is predicted to continue, with USSE capacity increasing 6 times more than wind energy capacity between 2020 and 2050. The prevalent technology of USSE development projects more than 1 megawatt (MW) that deliver energy to the electric transmission grid is expected to be photovoltaic [2]. Photovoltaic (PV) technology uses semiconductor cells to convert solar energy into electricity, and cells are assembled on panels that facilitate installation at energy facilities. Other types of technology, such as concentrating solar power, which uses reflected sunlight to generate thermal energy, is less common in the U.S., and development trends have moved from concentrating solar power to PV facilities [3].

The lack of carbon dioxide emissions generated from PV solar energy is a benefit to reducing the impact of climate change, which has been identified as the single largest threat to wildlife, including birds [4]. As with all forms of development, there are potential impacts from PV USSE development on birds, including habitat loss and potential for collision mortality [5]. Current PV technology requires approximately 1.5–3 hectares of land per MW of production, and vegetation is often removed in regions such as deserts in the southwestern U.S. [6]. However, the benefits of site restoration to pollinators and other wildlife has been recently recognized [7], and developers in some regions of the U.S. are moving towards ecologically-based site restoration and low impact site restoration [8]. Compared to impacts from wind energy development, direct impacts to birds from PV solar development are not well studied [5]. Only 1 paper in the peer-reviewed literature presents fatality information from a monitoring study at a PV solar facility in South Africa [9]. Other authors have summarized the potential effects of solar energy development [5] or have predicted the cumulative effects on birds from a projected solar buildout in the U.S. using PV and concentrated solar technology [6]. However, a current summary of bird fatalities at PV USSE facilities is generally lacking.

Given the rapid expansion of PV USSE, it is important to summarize the impacts to birds given their susceptibility to collide with anthropogenic structures so that the potential impacts of future PV USSE development can be evaluated [5,9]. Based on the comparatively sparse data available in the peer-reviewed literature, generalizations of direct impacts of PV USSE to birds are currently limited. For example, the unexpected detection of stranded, injured, or deceased water-associated birds (i.e., species that rely on water for foraging, reproduction, and/or roosting, such as herons and egrets [Pelecaniformes]) and water-obligate birds (i.e., species that cannot take flight from land, such as loons [Gaviiformes] and grebes [Podicipediformes]) at a PV USSE facility in the southwestern U.S. [5,10] led some researchers to propose that these groups of birds mistook a PV solar USSE for water (lake effect hypothesis) [10]. However, the extent of mortality of water-associated and water-obligate birds is unknown; indicating evidence supporting the lake effect hypothesis is in its infancy. Given the limited peer-reviewed papers available, it is unknown if the pattern of water-obligate birds at PV solar facilities is unique to one facility or widespread among facilities.

A potential source of information to enhance the understanding of bird fatality patterns at PV USSE facilities is the gray literature. Several fatality monitoring reports have been prepared voluntarily or to meet conditions included in the facility permit, and these reports contain important information that should be synthesized by presenting the information in one location. Similar gray literature has been synthesized to provide an understanding of bird mortality at wind projects [11,12]. Based on the relatively limited information on direct impacts to birds from PV USSE facilities, our objective involved searching the primary and gray literature to identify fatality studies in the U.S. that could be synthesized to provide inference into broader scale patterns in the region or regions represented by available studies. Specifically, we were interested in species composition and fatality estimates and how patterns varied spatially and temporally among facilities. Further, we compared fatality estimates from PV USSE reports we summarized to fatality estimates calculated by Walston et al. [6], whose analysis included multiple types of solar technology, including concentrating solar power.

## Methods

### Literature search

We used several sources of information to obtain studies on bird fatality rates at PV USSE. First, we conducted an internet-based search for studies on avian mortality at solar facilities in a manner similar to that of Walston et al. [6]. We used Google [13] and Web of Science [14] to search the term “solar energy” in various combinations with bird, avian, death, fatality, monitoring, mortality, and report. As fatality monitoring reports at PV USSE could also be available outside of the peer-reviewed literature, we obtained studies on standardized surveys for avian mortality at PV USSE from state and federal agencies, and from solar energy developers and operators. We excluded any studies of residential PV or studies of concentrated solar power or solar trough technologies.

### Data review

Guided by the study objectives, we examined each study to identify data that were appropriate for analysis. First, studies needed to use standardized fatality monitoring for a full year in the solar field. At a minimum, standardized monitoring must include searches for evidence of bird fatalities at regularly spaced intervals of time (although not necessarily consistent year round) at a fixed sample of the solar field at a PV USSE facility. Studies could include results from other infrastructure associated with PV USSE facilities (e.g., overhead lines, fences, generation-tie lines). Given that features such as power lines and fences are ubiquitous on the landscape, we only included data collected in the solar field since it represents the unique anthropogenic defining PV USSE facilities. For sites with multiple full study years, each year was treated as a separate study and is indicated by year in the analyses. Thus, we refer to each study as a site-year. We created acronyms for each site and site-year by Bird Conservation Region (BCR [15]), and distinguish among site-years when necessary (e.g., the first of two years of study at 1 site in BCR 33, Sonoran and Mojave Deserts, has acronym SMD1-1). BCRs are an appropriate rubric to group site-years because they were developed to aggregate ecologically similar regions in North America with similar bird communities, habitats, and resource management issues.

Carcass-level information, including species and date of discovery, was necessary for species and group composition and arrival phenology analyses. As not all birds die because of collision with infrastructure, studies could contain results for birds that were found alive (e.g., stranded, injured, dehydrated) in addition to carcasses. Thus, to capture all birds found with a single term, we refer to any discovery, regardless if the bird was alive, injured or deceased, as a detection. All detections found in the solar field, whether during standardized searches or incidentally, were included in species composition and phenology analyses. Authors of the original reports provided species determinations for detections, and we did not attempt to reclassify species or species groups. To understand the phenology of detection occurrence over the year, detections were assigned to Julian calendar days (1–365) and aggregated by week. We assigned seasons based on typical season dates used in monitoring reports from the southwestern U.S.: winter (November 1 –February 28/29), spring (March 1 –May 31), summer (June 1 –August 31), and fall (September 1 –October 31).

**Fig 1:**
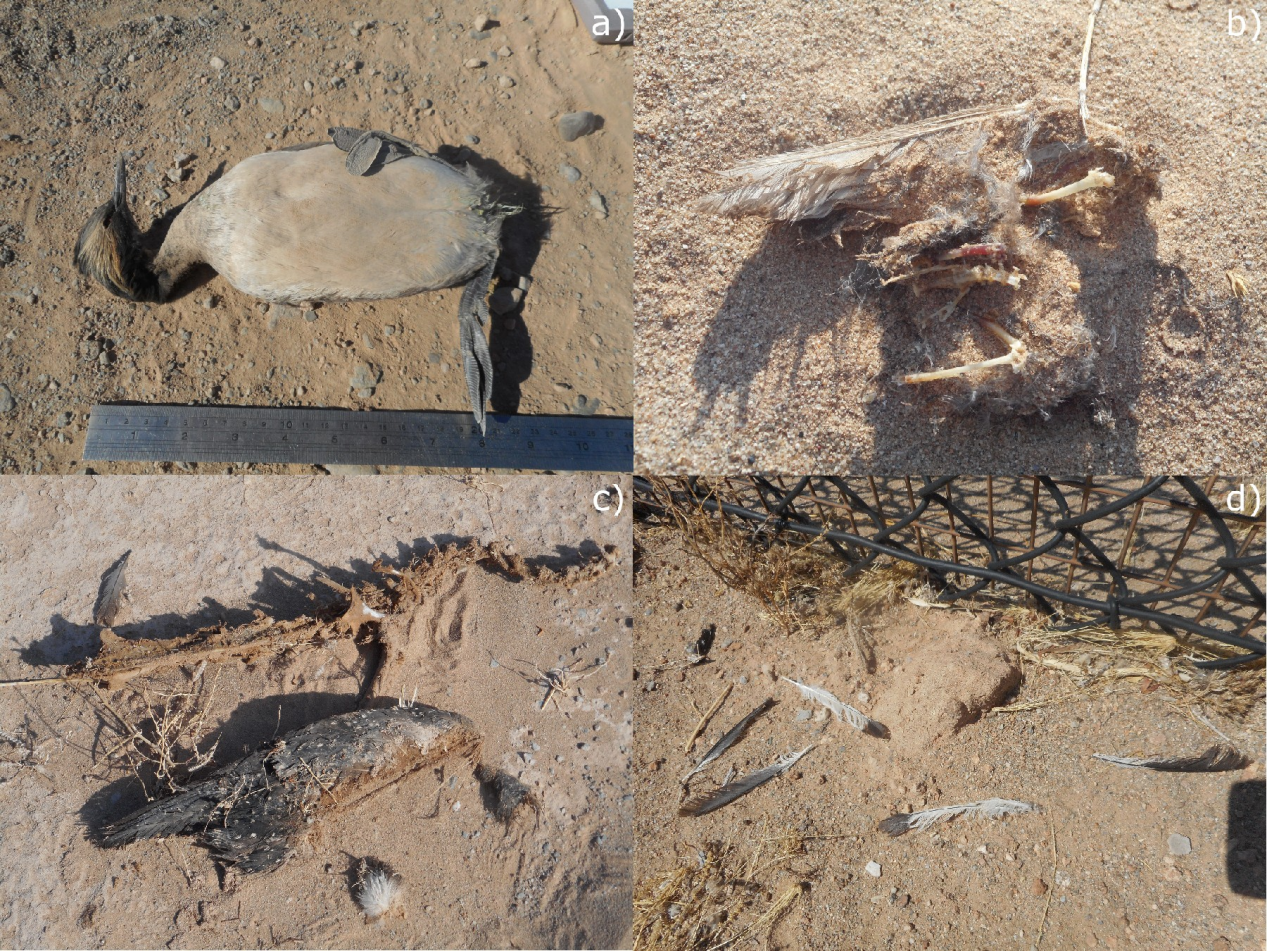
Examples of an intact carcass (a), partial carcass (b, c), and feather spot (d) found at PV USSE facilities in the Southwestern U.S.

Suspected cause of death is generally provided in fatality monitoring studies and could include collision with PV panels, overhead lines, or other infrastructure (e.g., buildings, fence lines), electrocution, predation, or an unknown cause [10]. Carcass condition was assigned by study authors using a variety of terms (e.g., whole-intact carcass, partial carcass, dead-fresh, dead-semi fresh) commonly used in fatality monitoring studies (e.g., U.S. Fish and Wildlife Service [16], Warren Hicks et al. [17]). We reclassified detections into 3 consistent categories based on the designations in the reports: partial (less than intact carcasses, with some bone or tissue present), feather spot (at least 5 tail feathers, or 2 primary feathers, or a total of at least 10 feathers with no attached bone or tissue, within 5 meters of each other [18]), and intact carcass or live find (Fig 1).

If fatality estimates were reported, the estimates were only used in our analyses if accompanied by a measure of variation (e.g., variance, standard deviation, or confidence interval) and if the estimates included adjustments for searcher efficiency and carcass persistence bias using a peer-reviewed fatality estimator (e.g., Huso [19]). To provide a comparison among site-years, we standardized fatality estimates, if not done so in the original study, by dividing the total fatality estimate by the nameplate capacity to calculate bird fatalities/MW/year. As there is also interest in understanding estimates in the context of land use and the potential contribution of background mortality (mortality not due to collision infrastructure [11]), we digitized the solar field for all sites from publicly available aerial imagery of facilities, and calculated the total area (in hectares) occupied by the solar field using ArcGIS [20]. We then divided the total fatality estimate by the measured hectares to obtain bird fatalities/hectare/year. To compare variability in estimates, we calculated a standardized confidence interval half-width as

$$0.5\;*\frac{upper\;confidence\;bound–lower\;confidence\;bound}{estimate}$$ (1)

to serve as an analog for coefficient of variation.

### Bird group categories

We used taxonomic order [21] or created groups based on a trait of interest (e.g., associated with water) to aggregate birds for analysis. Bird groups developed for this review are diurnal raptors, including eagles, hawks, kites, harriers, falcons, and vultures (Accipitriformes, Cathartiformes Falconiformes); and water-associated and water-obligate birds. We define water-associated birds (hereafter, water associates) based on life history traits, and include any species that relies primarily upon aquatic habitats for the purposes of foraging, reproduction, and/or roosting and could be present in the study areas based upon their known range. Water associates can walk on and take off from land. Given this definition, water associates include most species of ducks, geese, and swans (Anseriformes); pelicans, herons, ibises, bitterns, and allies (Pelecaniformes); coots and rails (Gruiformes); plovers, sandpipers, gulls, and allies (Charadriiformes); and osprey (*Pandion haliaetus*; Accipitriformes). We distinguish water-obligate birds (hereafter, water obligates), which rely on water for landing or take off, from water associates because of the importance of water obligates to the foundation of the lake-effect hypothesis. Water obligates include loons, grebes, cormorants (Suliformes), and diving ducks (Anseriformes), such as ruddy duck (*Oxyura jamaicensis*). S1 Appendix notes species that are considered water associates and water obligates.

In fatality monitoring reports, detections were identified with varying levels of resolution by the study authors, based on the condition of the detection, experience of the observer, and the degree to which the detection could be examined. The resolution of species identification in each site-year affected how detections were treated in the analyses. When detections could not be identified to individual species, but identified to a species group (e.g., unidentified duck), those detections were included in summaries by taxonomic order, as well as if a water associate or water obligate. Detections that could not be identified to a species group (e.g., unidentified large bird) were pooled into an “unidentified” category when comparing composition of different taxonomic orders, and aggregated in the category of “other birds” in analyses focused on water associates and water obligates.

### Fatality estimates

For each study included in the data summary of estimates, mathematical models known as estimators were used by the study authors to adjust the number of detections observed based on biases in the searchers’ abilities to find carcasses and scavenging/removal of the carcasses that might occur between searches. Searcher efficiency is a measure of how well an observer was able to find detections present in search plots, typically presented as a proportion of carcasses found to those available to be found during the trial. In the studies summarized, researchers placed trial bird carcasses to represent fatalities prior to actual searches to measure how many of the trials were found. To account for possible effects of bird size, small birds (100 grams or less on average), medium birds (101–999 grams on average), and large birds (1000 grams or greater on average) were used, with some studies using only small and large birds. Further, season was often included as a variable in analysis. Because observer detection is typically not perfect, searcher efficiency rates vary between 0 and 1.

Researchers measured how long a carcass persisted by conducting carcass persistence trials, during which they placed trial bird carcasses at the facility and checked on the status of the trial carcasses at varying intervals until the carcass vanished (assumed taken by a scavenger) or is not detectable due to weathering or decomposition or when the trial time period was over. The same size classes and seasonal estimates were included in carcass persistence analysis. Using estimates of searcher efficiency and carcass persistence, and the number of detections found during standardized searches, researchers estimated fatalities at each facility using a variation of the general underlying model:

$$F=\frac{c}{r*p*a}$$ (2)

where *F* is the total number of fatalities, *C* is the number of detections found and included in fatality estimation, *r* is the probability a carcass is available to be found on regular searches, *p* is the probability of detecting a carcass (given it is available), and *a* is the proportion of solar field surveyed [19,22].

Species, taxonomic order or group composition (i.e., the proportion of detections by species or group) is an important metric to understand how frequently species or groups occur in the detection dataset. However, species composition using the raw detection data could bias estimates because detection probability (i.e., probability a carcass is available to be found on a search, and detected by a searcher) differed between small, medium, and large carcasses. Generally, probability of detection tends to increase with carcass size. To properly account for detection probability among species, we calculated adjusted composition by dividing the detection counts by the product of searcher efficiency and carcass persistence probability (i.e., detection probability; as in Huso [19]). When detection bias estimates were reported, we calculated site-year specific adjusted composition, accounting for size class, season (as reported in each report), and year when applicable. Some studies did not distinguish between medium and large birds, in which case we used the large bird detection probability for medium-sized birds. When detection probability estimates were not reported for a site-year (4 site-years), we calculated adjusted composition using the average detection probability (by size class) based on all site-years with detection probabilities reported. Thus, the adjusted composition is a better representation of species composition than that calculated from raw detections.

### Comparison of fatality estimates with other studies

Walston et al. [6] produced a range of capacity-weighted average mortality rate estimates for Southern California and the United States, and based on fatality monitoring data from 3 studies in Southern California. These studies included 2 concentrating solar facilities, Solar One and Ivanpah Solar Electric Generating System, and one PV solar facility, California Valley Solar Ranch. Walston et al. [6] calculated a range of estimates from mortality attributable to the facility (2.7 birds/MW/year), mortality from unknown causes (7.3 birds/MW/year) and total mortality (9.9 birds/MW/year) at USSE facilities. When extrapolated to the built and planned solar capacity in the study area (6 gigawatts [GW]), the result was an estimated 16,200–59,400 bird fatalities/year. A new estimate of average annual per MW bird mortality for southern California was calculated using the PV solar (only) dataset presented in our study. In addition, we also extrapolated avian fatalities to all of California and Nevada, using updated solar buildout statistics from Walston et al. [8].

## Results

### Studies of bird fatality at PV solar facilities

**Fig 2:**
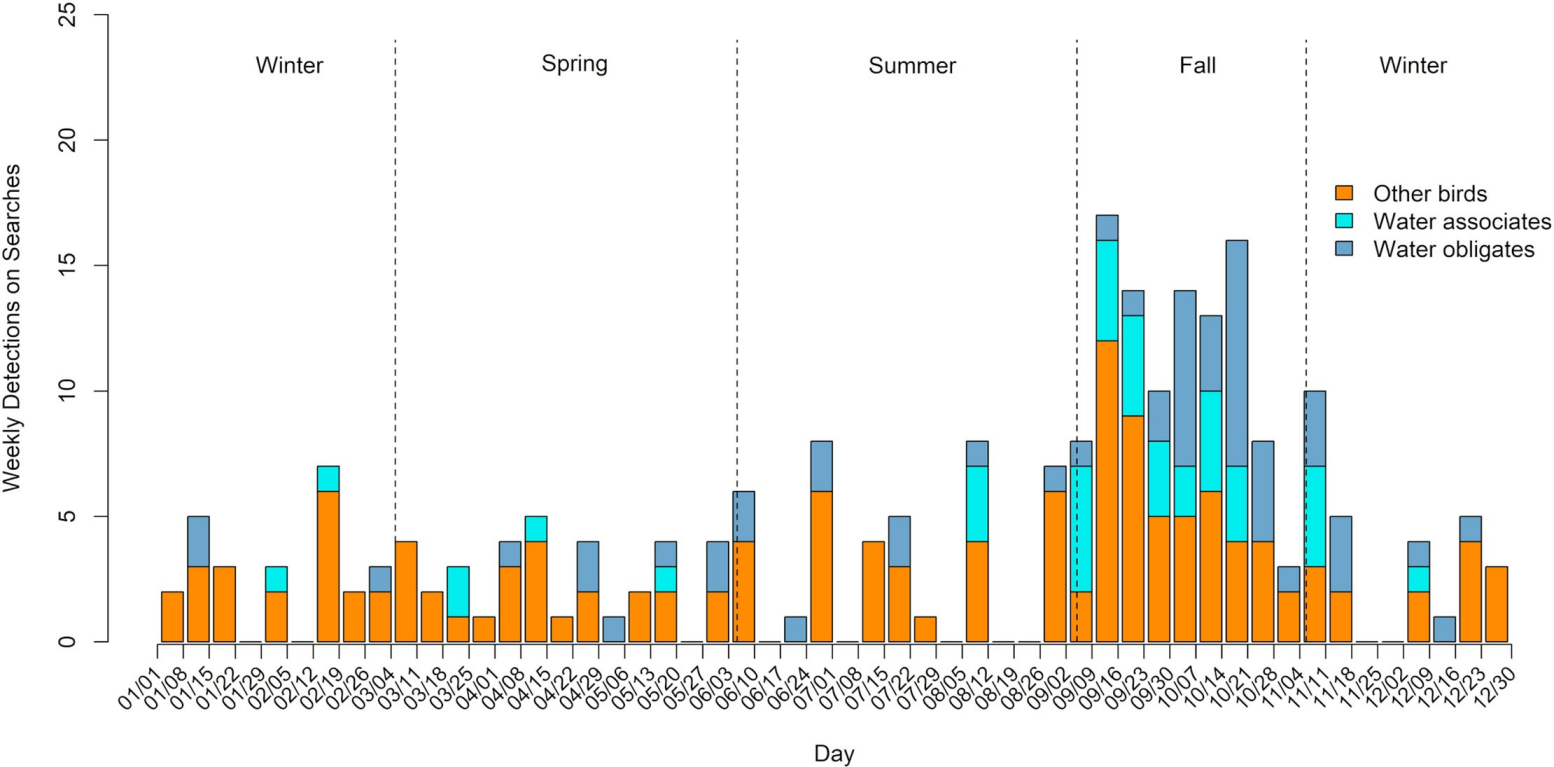
Phenology of avian detections found during standardized monitoring in the photovoltaic solar field at 7 sites from January 1, 2013, to September 1, 2018 in the Sonoran and Mojave Deserts and Great Basin Bird Conservation Regions. Water associates are species that rely on water for foraging, reproduction, and/or roosting; water obligates are species that cannot take flight from land; other birds are species not falling into either of those categories.

We identified useable data that met our inclusion criteria from 13 site-years occurring between November 2013 and September 2018 at 10 PV USSE facilities located in southern California in Imperial, Riverside, San Bernardino, and San Luis Obispo counties, and Nevada in Clark and Mineral counties (Fig 2; S2 Appendix). Facilities were located in the following BCRs: Sonoran and Mojave Deserts (SMD; BCR 33), Coastal California (CC; BCR 32), and Great Basin (GB; BCR 9). BCR 33 covers southeastern California and southern Nevada and adjoins the Sonoran Desert. The region is arid and dominated by cacti (Caryophyllales), slow-growing grasses (Poales), creosote bush (*Larrea tridentata*), and other desert shrubs. Waterbodies are relatively limited, and important bird resources include the Colorado River and the Salton Sea. BCR 32 extends from the coast of California inland to the foothills of the Sierra Nevada Mountains. Inland, the climate is hot and dry during summer, and the vegetation consists of mixed chaparral and remnant grasslands. BCR 9 is a relatively large area stretching from central Nevada north to southern British Columbia, Canada. The BCR is in the rain shadow of the Cascade Mountain Range, creating a dry climate with grasslands, sagebrush (Asterales), and shrub-steppe habitat in and lowlands, with piñon-juniper (*Pinus-Juniperus* spp.) woodlands and open ponderosa pine (*P*. *ponderosa*) forests at higher elevations.

Survey methods were relatively similar among the sites (S3 and S4 Appendices); however, project size varied from a nameplate capacity of 20 MW (SMD4) to 550 MW (SMD3 and CC2).

### Characterizing species and temporal patterns for bird fatalities at PV solar facilities

Across the 13 site-years in our dataset with a complete year of monitoring in the PV solar field, there were 669 avian detections. The data included detections of 86 identifiable species, representing 17 distinct taxonomic orders. The number of detections (unadjusted for detection probability) by site-year ranged from 6 (SMD5-1) to 274 (CC1-2). The total number of detections by species across all studies ranged from 1 (38 different identifiable species) to 145 (mourning dove [*Zenaida macroura*]; S1 Appendix). Songbirds (Passeriformes) and pigeons and doves (Columbiformes) had the highest number of detections (243 and 183, respectively), whereas hummingbirds (Apodiformes), woodpeckers (Piciformes), and cormorants had the fewest (2 in each taxonomic order).

One taxonomic order was found during all site-years, songbirds (Table 1). Doves and pigeons were also relatively widely represented, occurring at 62% (8 of 13) of site-years overall and at 60% (6 of 10) of site-years in the SMD BCR (Table 1). Water associates were not as widely distributed across site-years as water obligates; water associates occurred at 47% (6 of 13) of site-years, water obligates occurred at 77% (10 of 13) site-years. Water associates only occurred in site-years in the SMD BCR, whereas water obligates occurred in one of three site-years outside of the SMD BCR. Within the SMD BCR, water associates occurred at 57% (5 of 7) of facilities, and water obligates occurred at 100% (7 of 7) of facilities. Of identified species, there were no species common to all site-years; the most widely represented species was mourning dove, occurring at 62% (8 of 13) of site-years, including 60% (6 of 10) of site-years in the SMD BCR (S1 Appendix). The most common Passeriformes were western meadowlark (*Sturnella neglecta*), found at 54% (7 of 13) of site-years, and horned lark (*Eremophila alpestris*), found at 46% (6 of 13) of site-years; other species were found at 5 or fewer site-years. Of water obligates, only American coot (*Fulica americana*) and pied-billed grebe (*Podilymbus podiceps*) were found at a site-year outside of the SMD BCR (S1 Appendix). Among water obligates within the SMD BCR, common loon (*Gavia immer*) was found at 50% (5 of 10) of site-years; American coot and eared grebe (*P*. *nigricollis*) were each found at 40% (4 of 10) of site-years; other water obligates were found at fewer than 40% of site-years (S1 Appendix).

**Table 1:** Adjusted composition by taxonomic order (or group) by Bird Conservation Region (BCR) provided in fatality monitoring reports from January 1, 2013, to September 1, 2018. Data are presented by site-year so that SMD1-1 is the first year report for site SMD1 and SMD1-2 is the second year report for site SMD1.

	Sonoran and Mojave Deserts BCR										Great Basin BCR	Coastal California BCR		
Taxonomic Order Name or Group^a^	SMD1-1	SMD1-2	SMD2-1	SMD3-1	SMD3-2	SMD4-1	SMD5-1	SMD5-2	SMD6-1	SMD7-1	GB1-1	CC1-2	CC2-1	Total
**Cormorants and allies (Suliformes)**	0	0	0.64	0.53	0	0	0	0	0	0	0	0	0	0.09
**Cuckoos (Cuculiformes)**	7.38	2.17	3.18	0	0	0	0	0	0	0	0	0	5.85	0.68
**Doves and pigeons (Columbiformes)**	0	3.47	39.51	3.73	5.22	59.85	0	0.91	0	0	0	31.50	17.54	17.20
**Ducks and geese (Anseriformes)**	0	2.17	1.91	12.29	11.22	0	0	1.44	0	0	0	0	0	3.07
**Falcons and allies (Falconiformes)**	0	0	2.12	0	0	3.98	0	0	0	0	0	0	0	0.24
**Grebes (Podicipediformes)**	0	0.81	0	16.01	14.74	4.12	0	1.13	0	64.32	0	0.25	0	3.92
**Grouse and allies (Galliformes)**	0	2.17	0	1.05	0.77	6.30	0	0	0	0	0	0	17.53	0.79
**Raptors (Accipitriformes)**	0	0	0.64	0.81	0	2.27	0	0.53	20.07	13.85	5.80	0	0	0.76
**Loons (Gaviiformes)**	3.30	1.63	0	2.10	1.20	0	0	0	3.76	0	0	0	0	0.59
**Nightjars (Caprimulgiformes)**	31.36	0	2.15	0	4.82	0	21.55	0	0	0	0	0	0	1.52
**Owls (Strigiformes)**	0	0	5.31	0	0	4.12	0	0	0	0	0	1.25	0	0.95
**Pelicans and allies (Pelecaniformes)**	0	0	3.18	0	2.77	0	0	0	0	0	0	0	0	0.68
**Rails and allies (Gruiformes)**	0	0	4.88	14.62	17.68	0	12.47	3.49	0	0	0	0.5	0	4.79
**Shorebirds and gulls (Charadriiformes)**	0	0	2.15	1.29	3.72	0	0	0	0	0	0	0	0	0.82
**Songbirds (Passeriformes)**	57.96	79.15	2.15	34.68	26.07	14.97	65.98	83.20	25.39	21.83	91.78	64.98	35.45	54.71
**Hummingbirds (Apodiformes)**	0	1.93	0	1.46	0	0	0	0	0	0	0	0	0	0.31
**Unidentified**	0	6.51	32.20	10.42	11.78	4.39	0	7.56	50.78	0	2.42	1.52	23.63	8.58
**Woodpeckers (Piciformes)**	0	0	0	1.00	0	0	0	1.75	0	0	0	0	0	0.29
**Water associates**^**a**^	0	2.17	7.86	18.44	25.16	0	12.47	1.96	0	0	0	0	0	6.28
**Water obligates**^**a**^	3.30	2.44	4.88	28.40	26.18	4.12	0	4.63	3.76	64.32	0	0.75	0	7.75

^a^ Water associates are species that rely on water for foraging, reproduction, and/or roosting; water obligates are species that cannot take flight from land. Water-associates and water obligates (gray shaded rows) are groups composed of species from Orders and are not additive with Orders in the table. Water associates and water obligates do not contain the same species and are mutually exclusive.

Adjusted composition of songbirds was the highest percentage of all detections (54.71%) and the highest percentage of detections at 69.23% (9 of 13) of site-years (Table 1). Doves and pigeons were the next most highly represented taxonomic order, with adjusted composition of 17.20%, and the highest percentage of detections at 15.38% (2 of 13) of site-years. Although water associates and water obligates did not occur consistently across sites-years, resulting in a lower overall percentage of detections (6.28% and 7.75%, respectively), several site-years in the SMD BCR contributed more to the adjusted composition for water associates and water obligates across all site-years. Water associates composed 7.86%, 18.44%, 25.16%, and 12.47% of detections at SMD2-1, SMD3-1, SMD3-2, and SMD5-1, respectively; water obligates composed 28.4%, 26.18%, and 64.32% of detections at SMD3-1, SMD3-2, and SMD7-1, respectively. Overall, water associates composed 10.54% and water obligates had an adjusted composition of 12.62% in the SMD BCR, whereas these groups were absent from site-years in the GB BCR, and water obligates composed 0.75% in the CC BCR. Adjusted composition was higher for water associates and water obligates the closer the site was to the Salton Sea (Fig 2). The furthest sites from the Salton Sea showed almost no contribution of water associates and water obligates to be adjusted composition: GB1-1 (none), CC1-2 (0.75% water obligates), CC2-1 (none).

Identifiable species were most highly represented in adjusted composition by mourning dove (12.92%), horned lark (11.93%), house finch (*Haemorhous mexicanus*; 8.41%), and western meadowlark (7.78%; S1 Appendix). The high adjusted composition of mourning dove was driven by 3 site-years in the SMD and CC BCRs: SMD2-1, SMD4-1, and CC1-2. The water-obligate birds with the highest adjusted composition was American coot, representing 2.87% overall, and American coot was represented in higher percentages at 4 SMD BCR projects: SMD2-1 (4.25%), SMD3-1 (7.32%), SMD3-2 (9.47%), and SMD5-2 (3.49%). Eared grebe was reported in 4 site-years in the SMD BCR, although adjusted composition was less than 3% at all but 1 of those site-years. At SMD7-1, eared grebe composed 64.32% of all detections; however, there were only 7 total detections in the solar field for that site-year.

**Fig 3:**
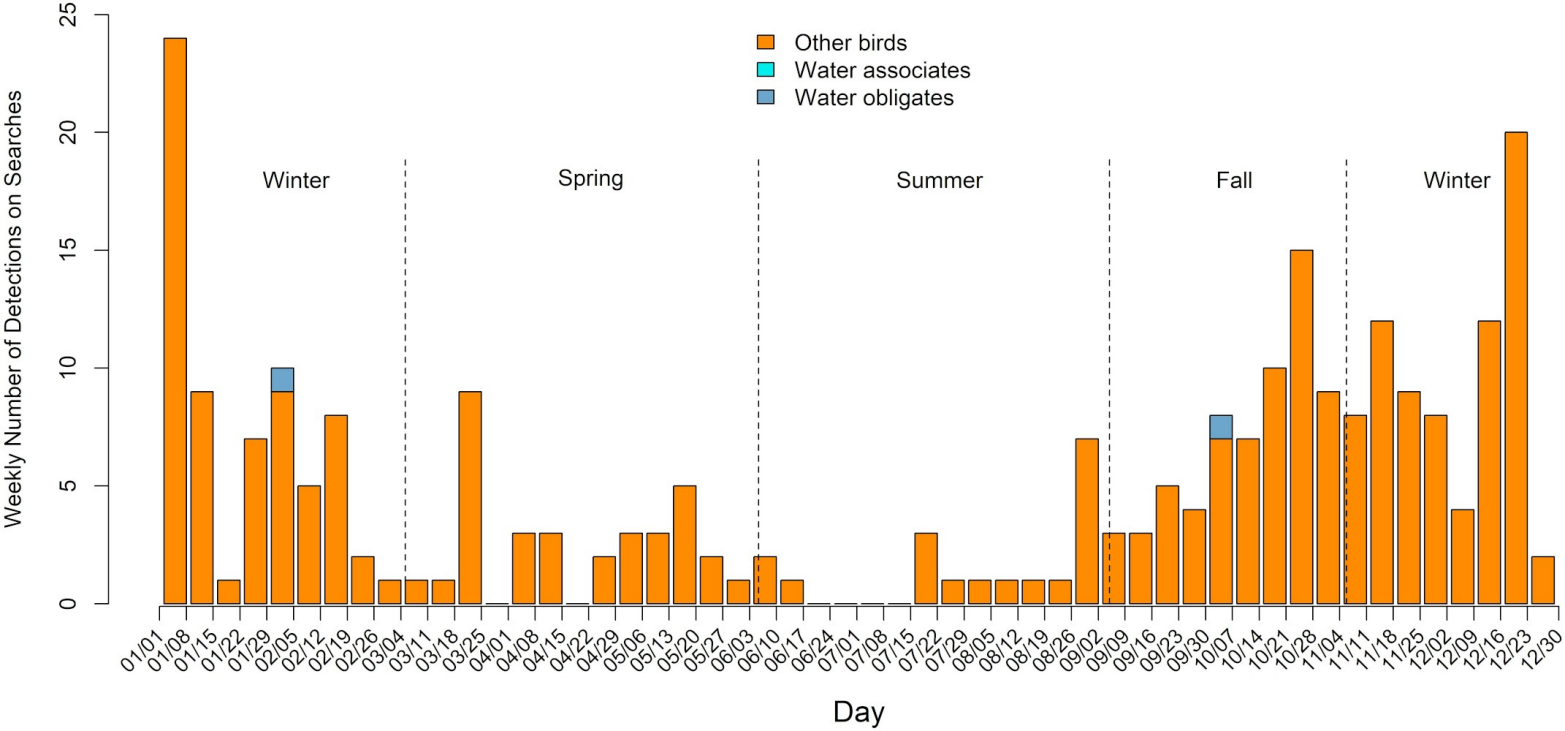
Phenology of avian detections found during standardized monitoring from January 1, 2013, to September 1, 2018 in the photovoltaic solar field at 2 sites in the Coastal California Bird Conservation Region. Water associates are species that rely on water for foraging, reproduction, and/or roosting; water obligates are species that cannot take flight from land; other birds are birds not falling into either of those categories.

**Fig 4:**
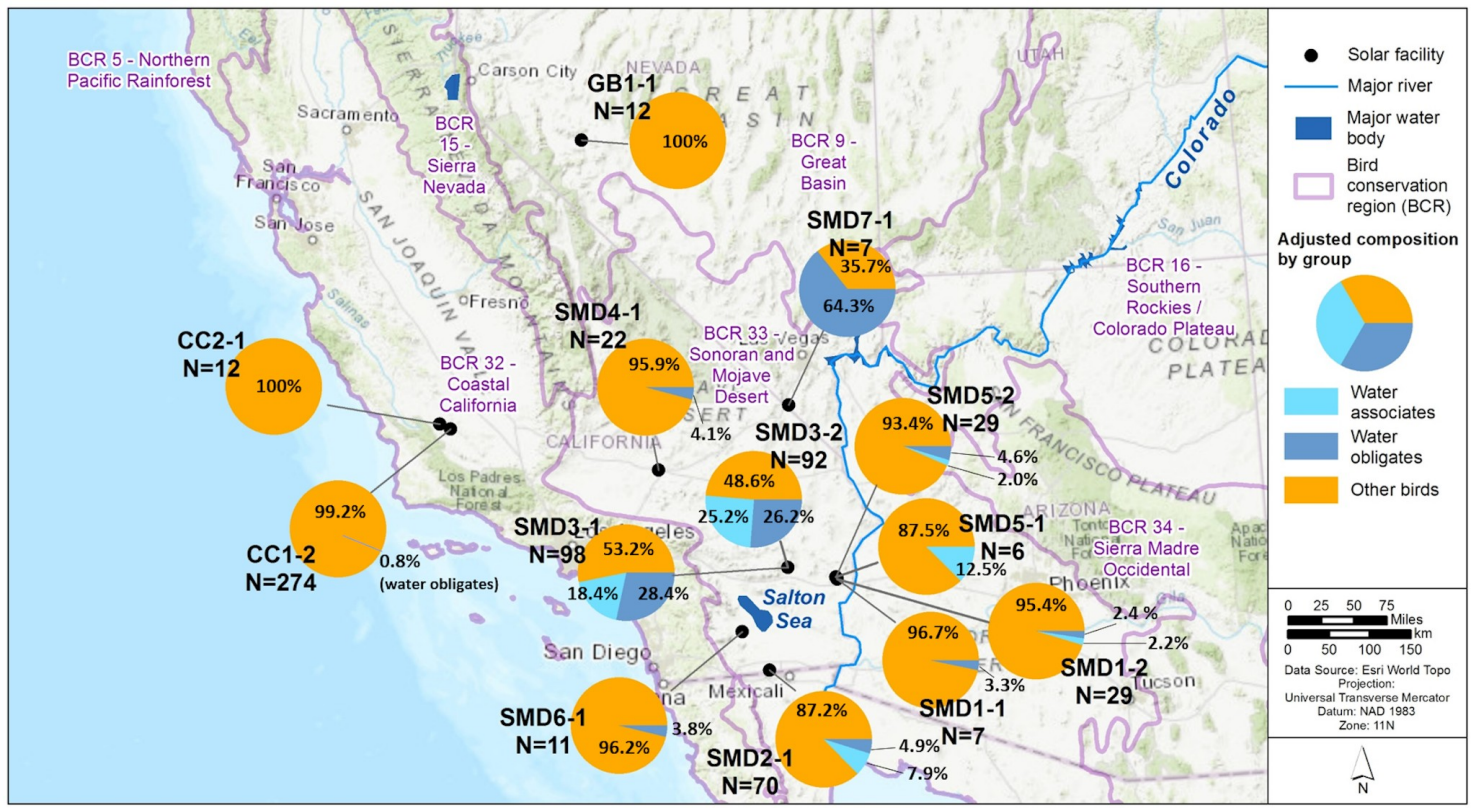
Adjusted composition of water obligates, water associates, and other birds for 13 fatality monitoring site-years at photovoltaic solar facilities in California and Nevada from January 1, 2013, to September 1, 2018. Water associates are species that rely on water for foraging, reproduction, and/or roosting; water obligates are species that cannot take flight from land; other birds are birds not falling into either of those categories. N = total number of detections for each site-year (e.g., CC2-1) represented on the map.

Timing of detections was included in all datasets except SMD2-1. The phenology of detections varied by season within the desert and non-desert ecoregions (Figs 3 and 4). For the SMD and GB BCRs, detections were found in all months of the year, and detections of water obligates were found in all months except February. Detections of water associates were found in all months except January, June, and July. The highest concentration of all birds, and water-associated and water-obligate bird detections in particular, was from September through early November at PV USSE facilities in the SMD and GB BCRs (Fig 3). In the CC BCR, the highest concentration of fatalities occurred between late September and early January, with only 2 water-obligate detections (September and January; Fig 4).

We summarized detections by day and week to attempt to capture variability in search schedules between studies for different site-years. The maximum number of detections by day and site-year for the CC BCR was 13 (CC1-2), and for the SMD BCRs the maximum number of detections in a single day was 5 (SMD3-1). The maximum number of detections by week—the most common search interval during the spring and fall survey periods in the dataset–and site-year was larger, with 23 for the CC BCR (CC1-2) and 9 for the SMD BCRs (SMD3-1 and SMD3-2).

### Characterizing carcass condition and suspected cause of death for bird fatalities at PV solar facilities

Carcass condition and suspected cause of death data were present for all detections except those from SMD2-1 (all detections) and 11 detections from SMD6-1 that were missing suspected cause data. Of the 599 avian detections with carcass condition data, feather spots had the highest overall adjusted composition (53.79%), and were the majority of detections for 5 of the taxonomic orders reported (Table 2). Partial carcass was the second most highly represented condition (31.65%), and composed the majority of detections for all taxonomic orders where feather spots were not the most highly represented category. Intact carcasses and live finds made up 14.56% of all detections, and were not more than 46% of detections for any single taxonomic order. Less than 1% of detections (8 of 599) were found alive.

**Table 2:** Adjusted composition by taxonomic order and carcass condition for detections provided in fatality monitoring reports ranging from January 1, 2013, to September 1, 2018.

Common Order Name	Intact Carcass or Live Find^a^	Partial Carcass	Feather Spot
**Cormorants and allies (Suliformes)**	0	100	0
**Cuckoos (Cuculiformes)**	20.49	58.06	21.45
**Doves and pigeons (Columbiformes)**	5.42	10.00	84.58
**Ducks and geese (Anseriformes)**	13.25	72.52	14.23
**Falcons and allies (Falconiformes)**	0	0	100
**Grebes (Podicipediformes)**	17.63	63.37	19.00
**Grouse and allies (Galliformes)**	0	34.68	65.32
**Raptors (Accipitriformes)**	45.73	41.85	12.43
**Loons (Gaviiformes)**	35.16	64.84	0
**Nightjars (Caprimulgiformes)**	26.83	73.17	0
**Owls (Strigiformes)**	0	13.07	86.93
**Pelicans and allies (Pelecaniformes)**	0	100	0
**Rails and allies (Gruiformes)**	25.05	61.13	13.82
**Shorebirds and gulls (Charadriiformes)**	0	100	0
**Songbirds (Passeriformes)**	17.31	24.18	58.51
**Hummingbirds (Apodiformes)**	0	68.6	31.4
**Unidentified**	0	57.51	42.49
**Woodpeckers (Piciformes)**	0	76.78	23.22
**Overall**	14.56	31.65	53.79

^a^Live find includes birds that were injured or stranded but unharmed in the PV solar array.

There were 96 detections discovered as intact carcasses with suspected cause of death recorded, representing 9 taxonomic orders (Table 3). The overall majority (61% adjusted composition) of intact carcasses and the majority within each taxonomic order represented were recorded with unknown or indeterminable cause of death based on field evaluation. When suspected cause of death was determinable, collision with a panel or other solar infrastructure composed the highest percentage of carcasses with a known cause of death for all taxonomic orders.

**Table 3:** Adjusted composition of intact carcasses or live finds by suspected cause of death for detections provided in fatality monitoring reports ranging from January 1, 2013, to September 1, 2018.

Common Order Name	Collision–PV Panel^a^	Collision-Line	Collision-Other	Electrocution	Predation	Unknown
**Cuckoos (Cuculiformes)**	0.00	0.00	0.00	0.00	0.00	100.00
**Doves and pigeons (Columbiformes)**	5.77	0.00	31.75	0.00	0.00	62.48
**Ducks and geese (Anseriformes)**	14.05	0.00	0.00	0.00	0.00	85.95
**Grebes (Podicipediformes)**	7.16	0.00	0.00	0.00	0.00	92.84
**Raptors (Accipitriformes)**	0.00	0.00	0.00	0.00	0.00	100.00
**Loons (Gaviiformes)**	0.00	0.00	0.00	0.00	0.00	100.00
**Nightjars (Caprimulgiformes)**	50.00	0.00	0.00	0.00	0.00	50.00
**Rails and allies (Gruiformes)**	27.15	0.00	0.00	0.00	0.00	72.85
**Songbirds (Passeriformes)**	15.75	16.15	10.88	1.94	1.93	53.35
**Overall**	15.82	11.36	9.47	1.36	1.36	60.63

^a^ PV = photovoltaic

### Characterizing avian fatality estimates at PV solar facilities

Annual all bird fatality estimates adjusted for detection probability and search effort were available for 11 of the 13 site-years (unavailable for CC2-1 and SMD2-1). Fatality estimates were standardized relative to the nameplate MW capacity of each PV USSE facility, a common metric used in the analysis of avian fatalities from energy generation sources, especially wind energy. Estimates ranged from 0.08 birds/MW/year (0.031 birds/hectare/year; SMD7-1) to 9.26 birds/MW/year (5.170 birds/hectare/year; CC1-2), with a mean of 2.49 birds/MW/year (1.088 birds/hectare/year; Table 4). Excluding CC1-2, which could be considered an outlier in the dataset as 42.70% of the detections were unknown-cause mourning dove feather spots and the estimate was more than 1.5 times higher than the next highest estimate, the average annual fatality rate was 1.82 birds/MW (0.680 birds/hectare/year). Confidence intervals were presented in the report for each site-year when an estimate was presented, and all confidence intervals were 90% confidence intervals, with the exception of SMD6-1 (95% confidence interval). Site-years showed a comparatively wide range of variability, even when standardized by the magnitude of the estimate. As an analog to coefficient of variation (standard deviation/estimate), the standardized confidence interval half-width varied from 0.23 (CC1-2) to 1.25 (SMD1-1). Thus, the upper/lower ends of the confidence intervals were generally separated from the estimate by a distance between 0.23 and 1.25 times the estimate itself.

**Table 4:** Annual all bird fatality estimates, adjusted for detection probability and search effort, per megawatt nameplate capacity and per hectare (with confidence intervals), for 11 fatality monitoring studies at photovoltaic solar facilities in California and Nevada from January 1, 2013, to September 1, 2018.

Project Acronym	Year	Megawatts	Array Area (Hectares)	Technology	Analysis Detections	Fatalities/Megawatt (Confidence Interval^a^)	Fatalities/Hectare (Confidence Interval)
**CC1-2**	2013–2014	250	448	tracker	150	9.26 (7.56–11.86)	5.170 (4.223–6.625)
**GB1-1**	2017–2018	50	140	tracker	14	5.72 (1.52–14.68)	2.037 (0.541–5.227)
**SMD1-1**	2016–2017	235	681	tracker	2	0.20 (0.01–0.46)	0.062 (0.003–0.157)
**SMD1-2**	2017–2018	235	681	tracker	18	2.08 (0.94–2.90)	0.719 (0.326–0.999)
**SMD3-1**	2015–2016	550	1,206	fixed	74	1.05 (0.88–1.56)	0.480 (0.402–0.713)
**SMD3-2**	2016–2017	550	1,206	fixed	74	1.92 (1.47–2.57)	0.874 (0.671–1.173)
**SMD4-1**	2017–2018	20	51	tracker	22	2.55 (1.40–4.95)	1.000 (0.549–1.942)
**SMD5-1**	2016–2017	250	727	tracker	3	0.23 (0.04–0.49)	0.078 (0.015–0.169)
**SMD5-2**	2017–2018	250	727	tracker	20	2.99 (1.17–6.32)	1.028 (0.403–2.174)
**SMD6-1**	2017–2018	50	138	tracker	11	1.36 (0.74–3.54)	0.494 (0.269–1.286)
**SMD7-1**	2016–2017	250	635	tracker	7	0.08 (0.03–0.22)	0.031 (0.011–0.085)

^a^All confidence intervals are 90% confidence intervals, with the exception of SMD6-1, which presented a 95% confidence interval.

**Fig 5:**
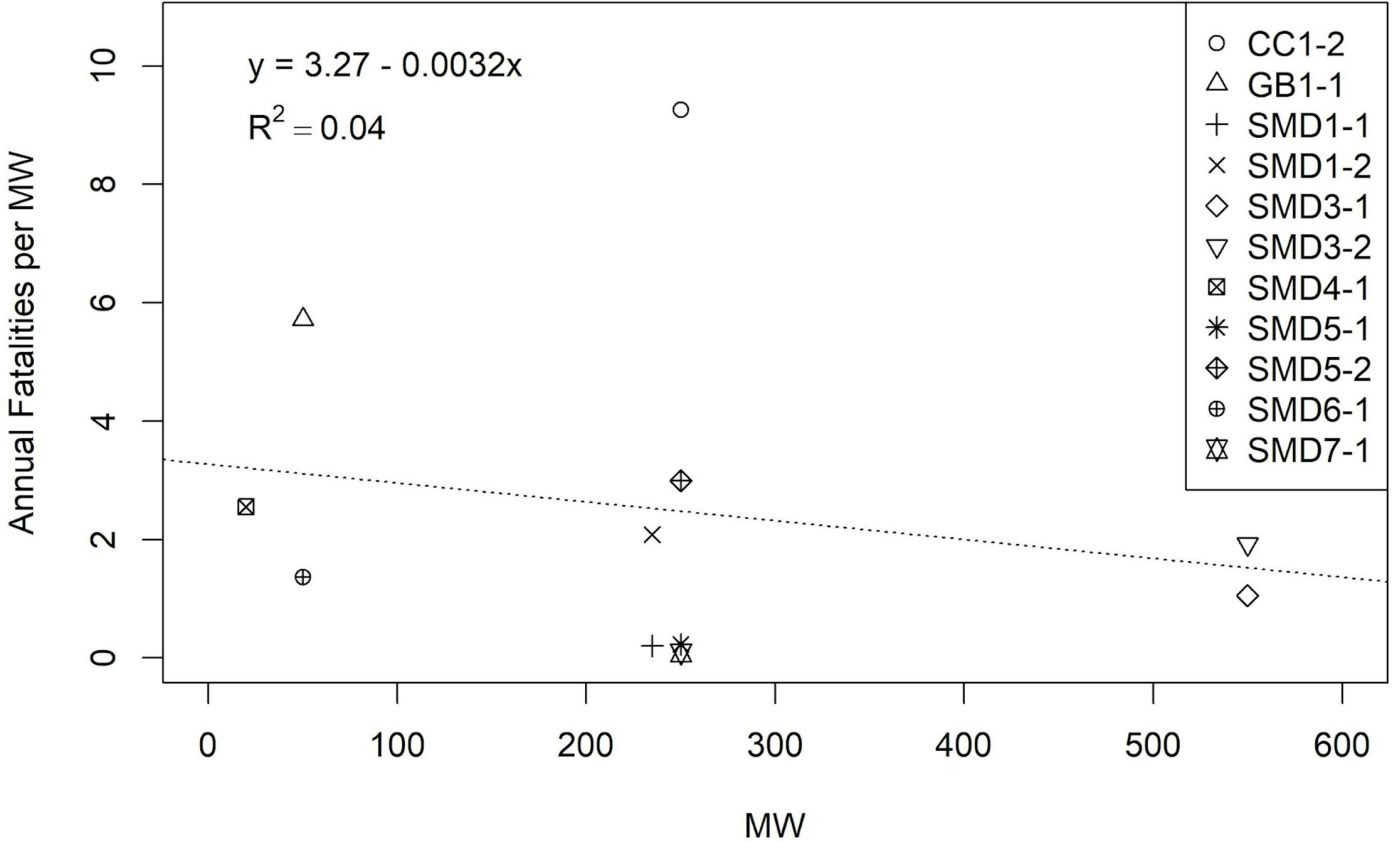
Annual all bird fatality estimates, adjusted for detection probability and search effort, per megawatt (MW; nameplate capacity), by nameplate capacity, from January 1, 2013, to September 1, 2018.

There was a strong positive correlation between nameplate MW capacity and solar field area (Pearson’s Correlation Coefficient, ρ = 0.97, p < 0.001), so we used nameplate MW capacity as the metric for facility size. Annual per MW fatality estimates showed a relatively weak, slightly negative relationship with facility size (slope = -0.003, p = 0.55, R^2^ = 0.04; Fig 5). CC1-2 was an outlier, but excluding these data did not appreciably change the overall relationship between fatality rate and facility size.

**Fig 6:**
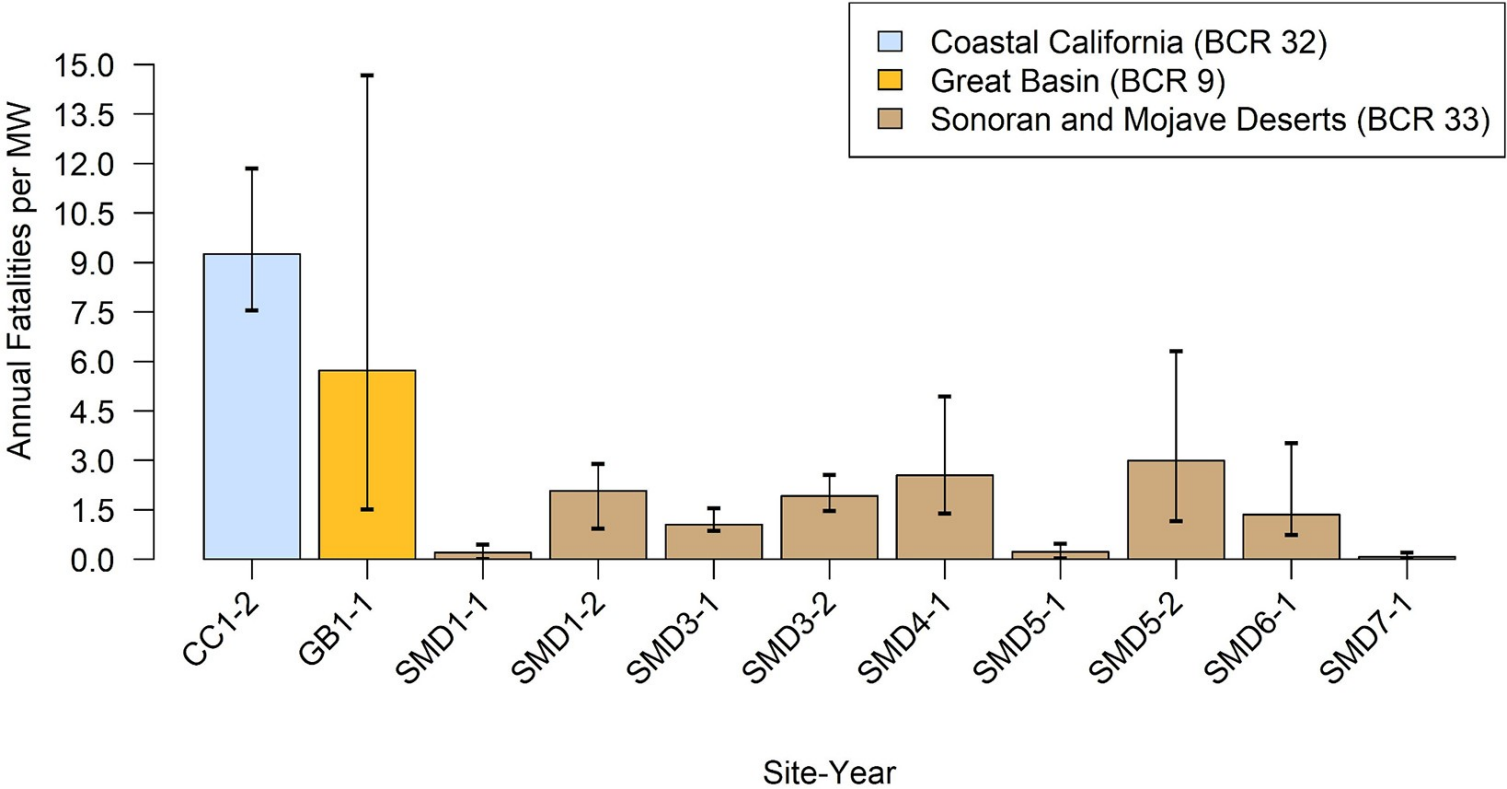
Annual all bird fatality estimates, adjusted for detection probability and search effort, per megawatt (nameplate capacity) by Bird Conservation Region, from January 1, 2013, to September 1, 2018. Vertical bars show 90% confidence interval around estimates for each study except SMD6-1, which only reported a 95% confidence interval.

The dataset was dominated by site-years in the SMD BCR, which showed variability in annual fatality rates between 0.08 and 2.99 birds/MW/year (Fig 6). Annual fatality rate estimates in the CC and GB BCRs, represented by 1 site-year each, were higher than the SMD BCR estimates. However, the estimate associated with GB1-1 had the widest 90% confidence interval of any site-year, extending below several estimates from the SMD BCR, and above the upper confidence bound of the estimates from CC1-2.

### Comparison of fatality estimates with other studies

Based on the dataset assembled for this manuscript, we updated the solar fatality estimate from Walston et al. [6] to reflect a larger dataset of estimates derived from systematic monitoring studies at PV USSE facilities that compose 9.7% of current and planned solar buildout in California and Nevada. We calculated the average annual fatality estimate of known and unknown cause per MW at PV USSE facilities in desert BCRs to be 1.82 birds/MW/year. Using the 6-GW capacity cited in Walston et al. [6], and assuming predominantly PV development, the result is an estimate of 10,920 fatalities/year in southern California, compared to Walston et al.’s [6] known and unknown cause estimate of 59,400 fatalities/year in southern California. Including the estimate from CC1-2 raises the average fatality rate to 2.49 birds/MW/year, or 14,940 bird fatalities/ year in Southern California. We also took updated estimates of USSE development in California (14.562 GW) and Nevada (2.458 GW) from Walston et al. [8] to derive an updated range of known and unknown cause estimates for all of California and Nevada: 30,976 (excluding CC1-2) to 42,193 (including CC1-2) bird fatalities per year.

## Discussion

Our study provided 4 main findings. First, we found variability in the distribution of taxonomic orders and species among and within BCRs; however, 3 species (mourning dove, horned lark, and western meadowlark) were consistently found among site-years, with adjusted composition greater than 5% over all site-years. Second, a phenology pattern emerged where most detections occurred in fall in the SMD BCR, with a pattern of higher detections occurring through winter in the CC BCR. Third, we found that most detections were of feather spots, and that most detections were attributed to an unknown cause of fatality. Last, we found that annual fatality rates never exceeded 2.99 fatalities/MW/year (1.03 fatalities/hectare/year) in the SMD BCR, were highest in the CC BCR where the rate was 9.26 fatalities/MW/year (5.17 fatalities/hectare/year), and that fatality rates did not correlate with nameplate capacity. Overall, the fatality rate we calculated for PV USSE using known and unknown cause fatalities was lower than that calculated by Walston et al. [6], who used multiple types of USSE development to generate an average annual fatality rate per MW. Taken together, our results offer important insight into the patterns of bird mortality at PV USSE facilities that will assist interested stakeholders in understanding the effects of an energy technology that is becoming more common on the landscape.

### Species composition and variation among PV solar facilities

We found variability in the species composition among site-years among and within a BCR. Songbirds occurred in all site-years, which is consistent with patterns at other anthropogenic features where songbirds were widely represented in fatality studies (e.g., Erickson et al. [11], Longcore et al. [23]). The species detected in the most site-years included mourning dove, western meadowlark, and horned lark. These 3 species, along with house finch, also had the highest adjusted composition across the dataset. Mourning dove, western meadowlark, and horned lark share several traits, including that these species are primarily ground dwelling, inhabit landscapes with relatively low-growing vegetation, and have comparatively large populations in the U.S. in regions where the studies occurred [24]. Mourning dove and house finch share a trait in that they associate with anthropogenic structures [24]. According to the Partners in Flight Bird Population Database [25], there are an estimated 12.8 million mourning doves, 13.8 million western meadowlarks, 16.09 million horned larks, and 14.2 million house finches in the 3 BCRs represented by site-years. Thus, the overall most common species found as detections are generally abundant in the regions where the studies occurred and the species share behavioral traits in that they move at or near ground level or associate with anthropogenic structures. It is possible that PV USSE facilities provide structure and an environmental microclimate that attracts birds and other species, but none of the studies we reviewed compared mortality data to live bird count data, so it is unknown if mortality at PV USSE facilities is associated with increased localized use.

We found that water associates were not as widely distributed among site-years as water obligates, and that water obligates occurred at 9 of 10 site-years in the SMD BCR and at 1 of 3 site-years in the CC and GB BCRs. Mortality of water associates and water obligates is known from other anthropogenic features, including wind turbines [26], communication towers [23,27], and buildings [28]. However, wind turbines, communication towers, and buildings represent comparatively tall, vertical hazards to migrating individuals, whereas PV solar panels at the sites are generally within 3 meters of the ground. The collision of water obligates with relatively low-lying PV panels raised questions by Kagan et al. [10] about the causal mechanism for occurrence (e.g., lake-effect hypothesis). None of the studies included in our summary provided hypotheses for the occurrence of water obligates, nor did the studies collect data to investigate potential causal mechanisms such as the amount of polarized light reflected by the PV panels or behavioral responses of water obligates to PV panels. Thus, none of the studies provide insight into the causal mechanism responsible for the presence of water obligates at PV USSE in the SMD BCR, and we avoid speculating about possible causes given the relative lack of important information (e.g., how water obligates perceive polarized light reflected from PV solar panels). Rather, we focus our review on summarizing the spatial and temporal patterns of occurrence.

The Salton Sea and Gulf of California serve as stop-over and winter habitat for hundreds of thousands of water associates and water obligates [29]. For example, the majority of the eared grebe population in the U.S. winters in the Gulf of California [29]. Thus, at a broad scale among BCRs, the concentration of water obligates in the SMD BCR at the Salton Sea is a plausible explanation for the variability in occurrence as concentrations of water obligates at similar stop-over areas are not known in the CC or GB BCRs near the sites. However, finer resolution spatial exposure data are needed to begin to understand variability among site-years within the SMD BCRs. Therefore, variation among BCRs appears associated with an abundance of migratory and over-wintering water obligates at the Salton Sea and the proximity of the sites to the Salton Sea, but we cannot readily interpret the variation among site-years within the SMD BCR given the absence of local exposure data at each site. An important limitation of our study and interpretation of the broad scale patterns of water-obligate bird occurrence is that our results are not predictive outside of the vicinity of the sites included. Our statements should not be interpreted as evidence there will be water-obligate bird mortality at PV USSE facilities developed in areas with concentrations of migrating or overwintering water obligates because the causal mechanism for fatality risk is unknown. Rather, additional fatality data collected can be evaluated to determine if results from a site align with, or fall outside of the pattern evident in our summary.

### Phenology

Peak number of detections per survey period was highest in fall in the Sonoran and Mojave Deserts and GB BCRs and highest in fall and winter in the CC BCR. The phenology of detections mirrors patterns found at other anthropogenic features (e.g., buildings), and coincides with higher abundance of birds following the breeding season [30]. The peak in detections in the fall season at site-years in the SMD and GB BCRs is likely influenced by an increase in the number of water associates and water obligates during the fall season. Although all bird detections tend to increase at the beginning of the fall period in September, detections of water associates and water obligates continued to increase until the end of October, whereas detections of other birds declined steadily throughout the fall. Waterfowl, loons, and grebes are all known to move in comparatively large numbers in fall when weather conditions are favorable for migration [31,32,33]. Eared grebes stage at the Great Salt Lake in Utah, and synchronize migration with upwards of 100,000 to 200,000 birds departing simultaneously en route to the Gulf of California [34]. Thus, the increase in water associates and water obligates during fall is likely explained by migratory movements to the wintering grounds.

Unlike mortality events of migratory birds at comparatively tall anthropogenic structures where hundreds of whole-intact carcasses have been found in a single night at a single communication tower or building [26], the highest number of carcasses detected in a site-year single visit was 13. Comparatively large-scale mortality events at communication towers or buildings are generally associated with lighting and nights with relatively low cloud ceilings [35,36]. In addition, no comparatively large downing events of water obligates were documented at any site-year, although relatively large downing events of eared and western (*Aechmophorus occidentalis*) grebes have been documented during poor weather, or associated with other factors such as wet parking lots [37]. Thus, the absence of large-scale mortality events of nocturnal migrants at PV USSE is likely best explained by the low stature of PV panels and the general lack of lighting.

### Carcass type, condition, and uncertainty

The majority of detections in the site-years were feather spots, and a cause of mortality could not be attributed for most detections, in contrast to patterns at comparatively tall structures where cause is typically attributed to collision (e.g., Erickson et al. [26], Loss et al. [28]). Feather spots could occur from a number of sources, including background mortality (e.g., mortality from predation [11]). At CC1-2, where the majority of detections were mourning doves, a prey species, fatality monitoring was conducted at reference plots outside of the solar facility. Reference plots equal in size to sample units within the facility (referred to as “tracker units”) at CC1-2 were searched concurrently with sample units in the solar field. The resulting adjusted fatality rate of 1.73 birds/tracker unit/year converts to an annual reference plot fatality rate of 6.92 birds/MW/year (based on the approximate MW to tracker unit ratio of 0.25 MW per tracker unit at the facility). All detections found in reference plots at CC1-2 were feather spots, primarily composed of Columbiformes (45%), supporting the idea that some proportion of the detections in the solar field at CC1-2 could be predation related, but the proportion cannot be conclusively stated because fatality events were not observed. At SMD3, Kagan et al. (2014) estimated from opportunistic necropsies 31% of the carcasses examined were likely impact trauma (e.g., collision with a solar panel or overhead line), 24% likely predation, with most of the remaining carcasses of unknown cause. Thus, when considering impacts to birds in general from anthropogenic structures, it is important to distinguish the certainty in cause for building and tower mortality, where intact carcasses are found below the structures, from PV USSE, where the majority of the detections are feather spots or partial carcasses found throughout the PV array field.

### Comparison of fatality estimates with other studies

We calculated an average annual fatality estimate for known and unknown cause per MW at PV USSE (2.49 fatalities/MW/year, upper estimate) that was less than that provided by Walston et al. [6] (9.9 fatalities/MW/year), and the difference is driven by 3 factors. First, Walston et al. [6] included data from the California Solar One and Ivanpah Solar Electric Generating System (Ivanpah) where the concentrating solar flux has been shown to singe migratory birds in fall and spring, and singeing does not occur at PV USSE [38]. Second, the fatality estimates Walston et al. [6] used in their analysis included all infrastructure monitored (e.g., overhead lines, generation tie-lines, fences), not just the solar field. Third, the only PV USSE facility included in Walston et al. [6] is CC1-2 in our study, which had an average annual fatality estimate 5.1 times higher than the average among the other 12 site-years, possibly due to the contribution of background mortality of mourning doves. Therefore, the average annual fatality estimate produced by Walston et al. [6] contains a mixture of solar technologies, was not limited to the PV solar field, and includes one PV facility with the highest annual fatality estimate among site-years. Thus, our average annual fatality estimate (2.49 birds/MW/year) reflects current PV USSE development. However, as CC1-2 is included in our analysis, an average annual fatality estimate of 1.82 birds/MW/year might be a more accurate representation at PV USSE facilities in the BCRs where the studies occurred.

Even our conservative average annual fatality estimate (2.49 birds/MW/year) of known and unknown detections was approximately 75% less than the known and unknown cause average annual fatality estimate from Walston et al. [6] based on a 6-GW capacity in southern California. Furthermore, our conservative average annual fatality estimate for the entirety of California and Nevada, based on updated capacity, was 29% less than the average annual fatality estimate by Walston et al. [6] for southern California alone. However, our conclusions from an expanded dataset align with Walston et al. [6] in that avian mortality at PV USSE was lower than other sources of anthropogenic bird mortality (e.g., Loss et al. [28]). The conclusions we reached are relevant for regions within the BCRs represented by our dataset, in particular the SMD BCR where most of the site-years occurred. Given that mortality risk is not well understood in different habitat contexts, we do not recommend extrapolating the average annual fatality estimates we calculated out to the current and projected buildout of the U.S., or to other BCRs with markedly different habitats (e.g., BCR 19, Central Mixed Grass Prairie).

## Conclusions

There are consistent patterns in several aspects of our analysis that could provide insight into potential patterns of bird mortality at PV USSE outside of the BCRs where the studies occurred; however, a primary limitation of our study in reaching broader generalizations is that 77% (10 of 13) of site-years occurred in the SMD BCR. Four patterns that could provide broader inference to other regions are: 1) the most widely occurring species among site-years have populations in the millions in the BCRs where studies occurred, and 3 of the top 4 species detected are ground-dwelling birds; 2) most detections occurred in fall; 3) there was no evidence of a comparatively large-scale fatality events of nocturnal migrating passerines or migrating water associates or water obligates; 4) most detections were of unknown cause feather spots. As none of the studies investigated the potential causal mechanism responsible for the occurrence of water obligates, generalizations are limited to mortality patterns in the SMD BCR where water obligates were found at 90% of site-years and 100% of PV USSE facilities. Proximity to a stop-over site for hundreds of thousands of water associates and water obligates could be a contributing factor to the variability among BCRs. The overall average annual fatality estimate can be generalized to the habitats in the BCRs where the studies occurred with more inference from the SMD BCR; however, generalizing the average annual fatality estimate in BCRs where studies did not occur is not appropriate. The intent of our summary was to provide an understanding of overarching patterns in bird mortality at PV USSE and we feel providing management recommendations is outside of the scope of our summary. Instead, we suggest that if fatality monitoring is conducted in areas outside of the regions where the studies occurred that researches evaluate their fatality patterns against our summary. In order to predict whether water-associated and water-obligate birds will occur at PV USSE outside of the SMD BCR, studies investigating the underlying causal mechanisms are needed. Further, a summary or additional studies of the potential contribution of background mortality to PV USSE fatality estimates could be considered to determine if suitable information exists to untangle facility-related from background mortality.

## Supporting information

S1 Appendix Adjusted composition and total detections of species by site year and Bird Conservation Region (BCR) provided in fatality monitoring reports ranging from January 1, 2013, to September 1, 2018. Data are presented by site-year so that SMD1-1 is the first year report for site SMD1 and SMD1-2 is the second year report for site SMD1.
(DOCX)

S2 Appendix Photovoltaic solar facility studies included in the dataset, with acronym and citation. (DOCX)

S3 Appendix Physical attributes and study information for photovoltaic solar facility studies included in the dataset. (DOCX)

S4 Appendix Study attributes for photovoltaic solar facility studies included in the dataset. (DOCX)

S5 Appendix Detection bias estimates for photovoltaic solar facility studies included in the dataset. (DOCX)

## References

[1] Wood Mackenzie, Limited. U.S. Solar Market Insight 2018 Q3; 2018 [cited 2019 October 17]. Available from: https://www.seia.org/research-resources/solar-market-insight-report-2018-q3

[2] U.S. Energy Information Administration. Annual energy outlook 2018 with projections to 2050; 2018 [cited 2019 October 17]. Available from: https://www.eia.gov/outlooks/aeo/pdf/AEO2018.pdf

[3] EDF Renewable Energy. Palen Solar Project CACA-48810 revised plan of development (formerly Palen Solar Power Project); 2017 [cited 2019 October 17]. Available from: https://eplanning.blm.gov/epl-front-office/projects/nepa/68122/123827/150984/0_Palen_Solar_Project_POD.pdf

[4] Urban MC. Accelerating extinction risk from climate change. Science. 2015; 348: 571–573. 10.1126/science.aaa4984 25931559

[5] Smith JA, Dwyer JF. Avian interactions with renewable energy infrastructure: an update. Condor 2016; 118: 411–423. 10.1650/CONDOR-15-61.1

[6] Walston LJ, Rollins KE, LaGory KE, Smith KP, Meyers SA. A preliminary assessment of avian mortality at utility-scale solar energy facilities in the United States. Renewable Energy. 2016; 92: 405–414. 10.1016/j.renene.2016.02.041

[7] Sinha P, Hoffman B, Sakers J, Althouse L. Best practices in responsible land use for improving biodiversity at a utility-scale solar facility. Case Stud Environ. 2018; 2(1): 1–12. 10.1525/cse.2018.001123

[8] Walston LJ, Mishra SK, Hartmann HM, Hlohowskyj I, McCall J, Macknick J. Examining the potential for agricultural benefits from pollinator habitat at solar facilities in the United States. Environ Sci Technol. 2018; 52: 7566–7576. 10.1021/acs.est.8b00020 29806456

[9] Visser E, Perlod V, Ralston-Paton S, Cardenal AC, Ryan PG. Assessing the impacts of a utility-scale photovoltaic solar energy facility on birds in the Northern Cape, South Africa. Renewable Energy. 2019; 133: 1285–1294. 10.1016/j.renene.2018.08.106

[10] Kagan RA, Viner TC, Trail PW, Espinoza EO. Avian mortality at solar energy facilities in southern California: a preliminary analysis. National Fish and Wildlife Forensic Laboratory. 2014; 28.

[11] Erickson WP, Wolfe MM, Bay KJ, Johnson DH, Gehring JL. A comprehensive analysis of small passerine fatalities from collision with turbines at wind energy facilities. PLoS ONE. 2014; 9: e107491 10.1371/journal.pone.0107491 25222738 PMC4164633

[12] Loss SR, Will T, Marra PP. Estimates of bird collision mortality at wind facilities in the contiguous United States. Biol Conserv. 2013; 168: 201–209. 10.1016/j.biocon.2013.10.007

[13] Google. Google search; 2019 [cited 2019 October 17]. Search engine [Internet]. Available from: https://www.google.com/

[14] Web of Science. Search on solar energy; 2019 [cited 2019 October 17]. Subscription-based citation indices [Internet]. Available from: https://clarivate.com/webofsciencegroup/solutions/web-of-science/

[15] Schmidt P, Myers G, Pashley D. A proposed framework for delineating ecologically-based planning, implementation, and evaluation units for cooperative bird conservation in the U.S. U.S. Fish and Wildlife Service. 1988 [cited 2019 October 17]. Available from: https://digitalmedia.fws.gov/digital/collection/document/id/214/

[16] U.S. Fish and Wildlife Service. Land-based wind energy guidelines; 2012 [cited 2019 October 17]. Available from: http://www.fws.gov/cno/pdf/Energy/2012_Wind_Energy_Guidelines_final.pdf

[17] Warren-Hicks W, Newman J, Wolpert R, Karas B, Tran L. Improving Methods for Estimating Fatality of Birds and Bats at Wind Energy Facilities; 2013 [cited 2019 October 17]. Available from: https://ww2.energy.ca.gov/2012publications/CEC-500-2012-086/CEC-500-2012-086.pdf

[18] California Energy Commission and California Department of Fish and Game. California guidelines for reducing impacts to birds and bats from wind energy development; 2007 [cited 2019 October 17]. Available from: https://tethys.pnnl.gov/sites/default/files/publications/Flint-2007.pdf

[19] Huso MM. An estimator of wildlife fatality from observed carcasses. Environmetrics. 2011; 22: 318–329. 10.1002/env.1052

[20] Environmental Systems Research Institute. ArcGIS Desktop. Release 10.2.1. Redlands, California: ESRI; 2013.

[21] American Ornithological Society. Checklist of North and Middle American birds. Checklist. [Internet]; 2019 [cited 2019 October 12]. Available from: http://checklist.aou.org/taxa/

[22] Huso MM, Dietsch T, Nicolai C. Mortality monitoring design for utility-scale solar power facilities. US Geological Survey; 2016 [cited 2019 October 17]. Available from: https://pubs.er.usgs.gov/publication/ofr20161087

[23] Longcore T, Rich C, Mineau P, MacDonald B, Bert D, Sullivan L, et al Avian mortality at communication towers in the United States and Canada: which species, how many, and where? Biol Conserv. 2013; 158:410–419. 10.1016/j.biocon.2012.09.019

[24] Cornell Lab of Ornithology. Bird guide; 2018 [cited 2018 December 9]. Guide [Internet]. Available from: https://www.allaboutbirds.org/guide/

[25] Partners in Flight. Population estimates database, version 3.0; 2019 [cited 2019 September 10]. Database [Internet]. Available from: http://pif.birdconservancy.org/PopEstimates

[26] Erickson WP, Johnson GD, Strickland MD, Young DP Jr., Sernka KJ, Good RE. Avian collisions with wind turbines: a summary of existing studies and comparisons to other sources of bird collision mortality in the United States. National Wind Coordinating Collaborative Resource Document; 2001. Available from: https://www.osti.gov/servlets/purl/822418

[27] Shire GG, Brown K, Winegrad G. Communication towers: a deadly hazard to birds; 2000 [cited 2019 October 17]. Available from: https://abcbirds.org/wp-content/uploads/2015/05/towerkillweb.pdf

[28] Loss SR, Will T, Loss SS, Marra PP. Bird–building collisions in the United States: estimates of annual mortality and species vulnerability. Condor. 2014 116: 8–23. 10.1650/CONDOR-13-090.1

[29] Shuford WD, Warnock N, Molina KC, Mulrooney B, Black AE. Avifauna of the Salton Sea: abundance, distribution, and annual phenology. Final report for EPA Contract R826552-01-0; 2000 [cited 2019 October 17]. Available from: https://nrm.dfg.ca.gov/FileHandler.ashx?DocumentID = 7312

[30] Drewitt A L, Langston RH. Collision effects of wind-power generators and other obstacles on birds. Ann N Y Acad Sci. 2008; 1134: 233–266. 10.1196/annals.1439.015 18566097

[31] Schummer M.L., Kaminski R.M., Raedeke A.H., and Graber D.A. Weather-related indices of autumn-winter dabbling duck abundance in middle North America. J Wildl Manage. 2010; 74: 94–101. 10.2193/2008-524

[32] Roberts AJ, Conover MR, Luft J, Neill J. Population fluctuations and distribution of staging eared grebes (*Podiceps nigricollis*) in North America. Can J Zool. 2013; 91: 906–913. 10.1139/cjz-2013-0181

[33] Frank MG, Conover MR. Weather and prey availability affect the timing of fall migration of eared grebes (*Podiceps nigricollis*) from Great Salt Lake. Wilson J Ornithol. 2017: 129: 98–111. 10.1676/1559-4491-129.1.98

[34] Williams AA, Laird NF. Weather and eared grebe winter migration near the Great Salt Lake, Utah. Int J Biometeorol. 2018; 62: 433–447. 10.1007/s00484-017-1452-8 29043451

[35] Larkin R. Investigating the behavioral mechanisms of tower kills In: Avian mortality at communication towers. Transcripts of the Proceedings of the Workshop on Avian Mortality at Communication Towers; 1999 8 11 (Vol. 11). New York: Cornell University, Ithaca; 2000.

[36] Gehring JL, Kerlinger P, Manville A II. Communication towers, lights, and birds: successful methods of reducing the frequency of avian collisions. Ecol Appl. 2009; 19: 505–514. 10.1890/07-1708.1 19323206

[37] Roberts AJ, Conover MR, Fusaro JL. Factors influencing mortality of eared grebes (*Podiceps nigricollis*) during a mass downing. Wilson J Ornithol. 2014; 126: 584–591. 10.1676/13-192.1

[38] Western EcoSystems Technology, Inc. 2017. Ivanpah Solar Electric Generating System avian & bat monitoring plan: 2015–2016 annual report; 2017 [cited 2019 October 17]. Available from: https://efiling.energy.ca.gov/Lists/DocketLog.aspx?docketnumber = 07-AFC-05C

