# Supplementary material for: A summary of bird mortality at photovoltaic utility scale solar facilities in the Southwestern U.S
Source: PLoS One. 2020 Apr 24;15(4):e0232034. doi: 10.1371/journal.pone.0232034 (PMC7182256; doi:10.1371/journal.pone.0232034)
Supplement: S1 Appendix — Data are presented by site-year so that SMD1-1 is the first year report for site SMD1 and SMD1-2 is the second year report for site SMD1. (DOCX) [file pone.0232034.s001.docx]

**S1 Appendix. Adjusted composition and total detections of species by site year and Bird Conservation Region (BCR) provided in fatality monitoring reports ranging from January 1, 2013 to September 1, 2018.** Data are presented by site-year so that SMD1-1 is the first year report for site SMD1 and SMD1-2 is the second year report for site SMD1.

|  | | | **Sonoran and Mojave Deserts BCR** | | | | | | | | | | **Great Basin BCR** | **Coastal California BCR** | |  | |
| --- | --- | --- | --- | --- | --- | --- | --- | --- | --- | --- | --- | --- | --- | --- | --- | --- | --- |
| **Species Name** | **Scientific Name** | **Taxonomic Order** | **SMD1-1** | **SMD1-2** | **SMD2-1** | **SMD3-1** | **SMD3-2** | **SMD4-1** | **SMD5-1** | **SMD5-2** | **SMD6-1** | **SMD7-1** | **GB1-1** | **CC1-2** | **CC2-1** | **Overall** | **Detections** |
| mourning dove | *Zenaida macroura* | Columbiformes | 0 | 3.47 | 11.68 | 3.08 | 0.58 | 22.15 | 0 | 0.91 | 0 | 0 | 0 | 30.25 | 5.85 | 12.92 | 145 |
| horned lark | *Eremophila alpestris* | Passeriformes | 0 | 19.52 | 0 | 1.87 | 0.84 | 0 | 0 | 0 | 0 | 0 | 54.84 | 19.71 | 35.45 | 11.93 | 54 |
| house finch | *Haemorhous mexicanus* | Passeriformes | 0 | 0 | 0 | 0 | 0 | 0 | 0 | 0 | 0 | 0 | 0 | 23.76 | 0 | 8.41 | 47 |
| western meadowlark | *Sturnella neglecta* | Passeriformes | 35.84 | 14.94 | 0 | 2.57 | 2.41 | 0 | 0 | 7.56 | 0 | 0 | 32.46 | 8.59 | 0 | 7.78 | 27 |
| unidentified bird (small) | NA | Unidentified | 0 | 6.51 | 32.2 | 5.62 | 3.25 | 0 | 0 | 7.56 | 50.78 | 0 | 0 | 1.52 | 23.63 | 6.83 | 31 |
| unidentified sparrow | NA | Passeriformes | 0 | 8.44 | 0 | 1.87 | 5.16 | 0 | 0 | 15.13 | 0 | 0 | 0 | 0 | 0 | 3.74 | 8 |
| American coot^ | *Fulica americana* | Gruiformes | 0 | 0 | 4.25 | 7.32 | 9.47 | 0 | 0 | 3.49 | 0 | 0 | 0 | 0.5 | 0 | 2.87 | 26 |
| unidentified grebe^ | NA | Podicipediformes | 0 | 0 | 0 | 9.88 | 14.01 | 0 | 0 | 0 | 0 | 0 | 0 | 0 | 0 | 2.75 | 26 |
| Savannah sparrow | *Passerculus sandwichensis* | Passeriformes | 0 | 0 | 2.15 | 2.75 | 1.38 | 0 | 0 | 3.78 | 0 | 0 | 0 | 3.54 | 0 | 2.3 | 13 |
| unidentified dove | *Columbidae genus* | Columbiformes | 0 | 0 | 21.47 | 0 | 2.41 | 0 | 0 | 0 | 0 | 0 | 0 | 0 | 0 | 2.15 | 11 |
| white-crowned sparrow | *Zonotrichia leucophrys* | Passeriformes | 0 | 0 | 0 | 0 | 0 | 0 | 0 | 15.13 | 0 | 0 | 0 | 0 | 0 | 1.92 | 2 |
| lesser nighthawk | *Chordeiles acutipennis* | Caprimulgiformes | 31.36 | 0 | 2.15 | 0 | 4.82 | 0 | 21.55 | 0 | 0 | 0 | 0 | 0 | 0 | 1.52 | 6 |
| Brewer's sparrow | *Spizella breweri* | Passeriformes | 0 | 5.78 | 0 | 1.29 | 3.25 | 0 | 0 | 1.89 | 0 | 0 | 0 | 0 | 0 | 1.45 | 7 |
| northern mockingbird | *Mimus polyglottos* | Passeriformes | 0 | 10.41 | 0 | 1.29 | 1.38 | 0 | 0 | 0 | 0 | 0 | 0 | 0 | 0 | 1.44 | 3 |
| unidentified warbler | NA | Passeriformes | 0 | 0 | 0 | 0 | 2.41 | 0 | 0 | 7.56 | 0 | 0 | 0 | 0 | 0 | 1.32 | 2 |
| rock pigeon | *Columba livia* | Columbiformes | 0 | 0 | 2.12 | 0 | 1.35 | 17.55 | 0 | 0 | 0 | 0 | 0 | 1.25 | 11.69 | 1.28 | 15 |
| unidentified bird (unknown size) | NA | Unidentified | 0 | 0 | 0 | 4.8 | 6 | 0 | 0 | 0 | 0 | 0 | 0 | 0 | 0 | 1.21 | 7 |
| sora* | *Porzana carolina* | Gruiformes | 0 | 0 | 0 | 4.62 | 6 | 0 | 0 | 0 | 0 | 0 | 0 | 0 | 0 | 1.2 | 7 |
| ash-throated flycatcher | *Myiarchus cinerascens* | Passeriformes | 0 | 10.41 | 0 | 0 | 0 | 0 | 0 | 0 | 0 | 0 | 0 | 0 | 0 | 1.15 | 1 |
| Say's phoebe | *Sayornis saya* | Passeriformes | 0 | 0 | 0 | 1.39 | 0 | 0 | 0 | 7.56 | 0 | 0 | 0 | 0 | 0 | 1.05 | 2 |
| unidentified teal* | *Anas spp* | Anseriformes | 0 | 2.17 | 0 | 1.63 | 4.46 | 0 | 0 | 0 | 0 | 0 | 0 | 0 | 0 | 1.01 | 8 |
| common raven | *Corvus corax* | Passeriformes | 6.43 | 0 | 0 | 2.54 | 0 | 0 | 0 | 0 | 0 | 21.83 | 0 | 1.8 | 0 | 0.97 | 18 |
| sage thrasher | *Oreoscoptes montanus* | Passeriformes | 0 | 0 | 0 | 0 | 0 | 0 | 0 | 7.56 | 0 | 0 | 0 | 0 | 0 | 0.96 | 1 |
| unidentified duck* | NA | Anseriformes | 0 | 0 | 0 | 4.45 | 4.27 | 0 | 0 | 0 | 0 | 0 | 0 | 0 | 0 | 0.93 | 10 |
| burrowing owl | *Athene cunicularia* | Strigiformes | 0 | 0 | 5.31 | 0 | 0 | 0 | 0 | 0 | 0 | 0 | 0 | 1.25 | 0 | 0.88 | 10 |
| loggerhead shrike | *Lanius ludovicianus* | Passeriformes | 0 | 0 | 0 | 3.75 | 0 | 0 | 0 | 0 | 0 | 0 | 0 | 1.52 | 0 | 0.79 | 5 |
| northern rough-winged swallow | *Stelgidopteryx serripennis* | Passeriformes | 0 | 0 | 0 | 1.39 | 0 | 14.97 | 0 | 0 | 25.39 | 0 | 0 | 0 | 0 | 0.69 | 4 |
| eared grebe^ | *Podiceps nigricollis* | Podicipediformes | 0 | 0.81 | 0 | 2.63 | 0 | 0 | 0 | 1.13 | 0 | 64.32 | 0 | 0 | 0 | 0.68 | 10 |
| greater roadrunner | *Geococcyx californianus* | Cuculiformes | 7.38 | 2.17 | 3.18 | 0 | 0 | 0 | 0 | 0 | 0 | 0 | 0 | 0 | 5.85 | 0.68 | 6 |
| Virginia rail* | *Rallus limicola* | Gruiformes | 0 | 0 | 0 | 2.68 | 2.21 | 0 | 12.47 | 0 | 0 | 0 | 0 | 0 | 0 | 0.66 | 5 |
| ruby-crowned kinglet | *Regulus calendula* | Passeriformes | 0 | 3.86 | 0 | 0 | 1.38 | 0 | 0 | 0 | 0 | 0 | 0 | 0 | 0 | 0.63 | 3 |
| western kingbird | *Tyrannus verticalis* | Passeriformes | 0 | 3.86 | 0 | 0 | 1.38 | 0 | 0 | 0 | 0 | 0 | 0 | 0 | 0 | 0.63 | 3 |
| common loon^ | *Gavia immer* | Gaviiformes | 3.3 | 1.63 | 0 | 2.1 | 1.2 | 0 | 0 | 0 | 3.76 | 0 | 0 | 0 | 0 | 0.59 | 13 |
| brown-headed cowbird | *Molothrus ater* | Passeriformes | 0 | 0 | 0 | 0 | 2.41 | 0 | 14.81 | 0 | 0 | 0 | 0 | 0 | 0 | 0.54 | 2 |
| Brewer's blackbird | *Euphagus cyanocephalus* | Passeriformes | 0 | 0 | 0 | 0 | 0 | 0 | 0 | 0 | 0 | 0 | 0 | 1.52 | 0 | 0.54 | 3 |
| yellow-rumped warbler | *Setophaga coronata* | Passeriformes | 0 | 0 | 0 | 2.57 | 0 | 0 | 0 | 0 | 0 | 0 | 0 | 1.01 | 0 | 0.53 | 4 |
| Eurasian collared-dove | *Streptopelia decaocto* | Columbiformes | 0 | 0 | 2.12 | 0 | 0 | 20.15 | 0 | 0 | 0 | 0 | 0 | 0 | 0 | 0.5 | 8 |
| Lincoln's sparrow | *Melospiza lincolnii* | Passeriformes | 0 | 0 | 0 | 0 | 0 | 0 | 0 | 3.78 | 0 | 0 | 0 | 0 | 0 | 0.48 | 2 |
| orange-crowned warbler | *Leiothlypis celata* | Passeriformes | 0 | 0 | 0 | 0 | 0 | 0 | 0 | 3.78 | 0 | 0 | 0 | 0 | 0 | 0.48 | 2 |
| Townsend's warbler | *Setophaga townsendi* | Passeriformes | 0 | 0 | 0 | 1.46 | 0.84 | 0 | 0 | 1.89 | 0 | 0 | 0 | 0 | 0 | 0.46 | 3 |
| Gambel's quail | *Callipepla gambelii* | Galliformes | 0 | 2.17 | 0 | 0 | 0.77 | 6.3 | 0 | 0 | 0 | 0 | 0 | 0 | 0 | 0.46 | 4 |
| red-tailed hawk | *Buteo jamaicensis* | Accipitriformes | 0 | 0 | 0 | 0 | 0 | 2.27 | 0 | 0 | 7.51 | 13.85 | 5.8 | 0 | 0 | 0.41 | 5 |
| unidentified bird (medium) | NA | Unidentified | 0 | 0 | 0 | 0 | 2.23 | 4.39 | 0 | 0 | 0 | 0 | 0 | 0 | 0 | 0.4 | 4 |
| California gull* | *Larus californicus* | Charadriiformes | 0 | 0 | 0 | 0 | 2.63 | 0 | 0 | 0 | 0 | 0 | 0 | 0 | 0 | 0.39 | 3 |
| western grebe^ | *Aechmophorus occidentalis* | Podicipediformes | 0 | 0 | 0 | 2.85 | 0.73 | 4.12 | 0 | 0 | 0 | 0 | 0 | 0 | 0 | 0.36 | 8 |
| least bittern* | *Ixobrychus exilis* | Pelecaniformes | 0 | 0 | 0 | 0 | 2.41 | 0 | 0 | 0 | 0 | 0 | 0 | 0 | 0 | 0.36 | 1 |
| summer tanager | *Piranga rubra* | Passeriformes | 0 | 0 | 0 | 0 | 2.41 | 0 | 0 | 0 | 0 | 0 | 0 | 0 | 0 | 0.36 | 1 |
| white-winged dove | *Zenaida asiatica* | Columbiformes | 0 | 0 | 2.12 | 0.65 | 0.88 | 0 | 0 | 0 | 0 | 0 | 0 | 0 | 0 | 0.35 | 4 |
| northern flicker | *Colaptes auratus* | Piciformes | 0 | 0 | 0 | 1 | 0 | 0 | 0 | 1.75 | 0 | 0 | 0 | 0 | 0 | 0.29 | 2 |
| ruddy duck^ | *Oxyura jamaicensis* | Anseriformes | 0 | 0 | 0 | 2.44 | 0.77 | 0 | 0 | 0 | 0 | 0 | 0 | 0 | 0 | 0.28 | 4 |
| domestic chicken | *Gallus gallus domesticus* | Galliformes | 0 | 0 | 0 | 0 | 0 | 0 | 0 | 0 | 0 | 0 | 0 | 0 | 17.53 | 0.27 | 3 |
| American pipit | *Anthus rubescens* | Passeriformes | 0 | 0 | 0 | 1.29 | 0 | 0 | 0 | 0 | 0 | 0 | 0 | 0.51 | 0 | 0.26 | 2 |
| western tanager | *Piranga ludoviciana* | Passeriformes | 0 | 0 | 0 | 0 | 0 | 0 | 21.55 | 0 | 0 | 0 | 0 | 0 | 0 | 0.26 | 1 |
| American kestrel | *Falco sparverius* | Falconiformes | 0 | 0 | 2.12 | 0 | 0 | 3.98 | 0 | 0 | 0 | 0 | 0 | 0 | 0 | 0.24 | 3 |
| hooded oriole | *Icterus cucullatus* | Passeriformes | 0 | 0 | 0 | 0 | 0 | 0 | 0 | 1.89 | 0 | 0 | 0 | 0 | 0 | 0.24 | 1 |
| MacGillivray's warbler | *Geothlypis tolmiei* | Passeriformes | 0 | 0 | 0 | 0 | 0 | 0 | 0 | 1.89 | 0 | 0 | 0 | 0 | 0 | 0.24 | 1 |
| vesper sparrow | *Pooecetes gramineus* | Passeriformes | 0 | 0 | 0 | 0 | 0 | 0 | 0 | 1.89 | 0 | 0 | 0 | 0 | 0 | 0.24 | 1 |
| yellow-headed blackbird | *Xanthocephalus xanthocephalus* | Passeriformes | 0 | 0 | 0 | 0 | 0 | 0 | 0 | 1.89 | 0 | 0 | 0 | 0 | 0 | 0.24 | 1 |
| green-winged teal* | *Anas crecca* | Anseriformes | 0 | 0 | 0 | 0 | 0.77 | 0 | 0 | 0.91 | 0 | 0 | 0 | 0 | 0 | 0.23 | 2 |
| Allen's hummingbird | *Selasphorus sasin* | Apodiformes | 0 | 1.93 | 0 | 0 | 0 | 0 | 0 | 0 | 0 | 0 | 0 | 0 | 0 | 0.21 | 1 |
| yellow-breasted chat | *Icteria virens* | Passeriformes | 0 | 1.93 | 0 | 0 | 0 | 0 | 0 | 0 | 0 | 0 | 0 | 0 | 0 | 0.21 | 1 |
| mallard* | *Anas platyrhynchos* | Anseriformes | 0 | 0 | 1.27 | 0.53 | 0.36 | 0 | 0 | 0 | 0 | 0 | 0 | 0 | 0 | 0.2 | 4 |
| common yellowthroat | *Geothlypis trichas* | Passeriformes | 0 | 0 | 0 | 0 | 0 | 0 | 14.81 | 0 | 0 | 0 | 0 | 0 | 0 | 0.18 | 1 |
| lark sparrow | *Chondestes grammacus* | Passeriformes | 15.68 | 0 | 0 | 0 | 0 | 0 | 0 | 0 | 0 | 0 | 0 | 0 | 0 | 0.18 | 1 |
| marsh wren | *Cistothorus palustris* | Passeriformes | 0 | 0 | 0 | 0 | 0 | 0 | 14.81 | 0 | 0 | 0 | 0 | 0 | 0 | 0.18 | 1 |
| European starling | *Sturnus vulgaris* | Passeriformes | 0 | 0 | 0 | 0 | 0 | 0 | 0 | 0 | 0 | 0 | 0 | 0.51 | 0 | 0.18 | 1 |
| house sparrow | *Passer domesticus* | Passeriformes | 0 | 0 | 0 | 0 | 0 | 0 | 0 | 0 | 0 | 0 | 0 | 0.51 | 0 | 0.18 | 1 |
| mountain bluebird | *Sialia currucoides* | Passeriformes | 0 | 0 | 0 | 0 | 0 | 0 | 0 | 0 | 0 | 0 | 0 | 0.51 | 0 | 0.18 | 1 |
| song sparrow | *Melospiza melodia* | Passeriformes | 0 | 0 | 0 | 0 | 0 | 0 | 0 | 0 | 0 | 0 | 0 | 0.51 | 0 | 0.18 | 1 |
| spotted towhee | *Pipilo maculatus* | Passeriformes | 0 | 0 | 0 | 0 | 0 | 0 | 0 | 0 | 0 | 0 | 0 | 0.51 | 0 | 0.18 | 1 |
| Swainson's thrush | *Catharus ustulatus* | Passeriformes | 0 | 0 | 0 | 0 | 0 | 0 | 0 | 0 | 0 | 0 | 0 | 0.51 | 0 | 0.18 | 1 |
| unidentified tern* | NA | Charadriiformes | 0 | 0 | 2.15 | 0 | 0 | 0 | 0 | 0 | 0 | 0 | 0 | 0 | 0 | 0.18 | 1 |
| red-shouldered hawk | *Buteo lineatus* | Accipitriformes | 0 | 0 | 0 | 0 | 0 | 0 | 0 | 0 | 12.56 | 0 | 0 | 0 | 0 | 0.18 | 2 |
| lesser goldfinch | *Spinus psaltria* | Passeriformes | 0 | 0 | 0 | 2.57 | 0 | 0 | 0 | 0 | 0 | 0 | 0 | 0 | 0 | 0.17 | 2 |
| red-winged blackbird | *Agelaius phoeniceus* | Passeriformes | 0 | 0 | 0 | 0 | 0 | 0 | 0 | 0 | 0 | 0 | 4.48 | 0 | 0 | 0.16 | 1 |
| unidentified gull* | NA | Charadriiformes | 0 | 0 | 0 | 0 | 1.09 | 0 | 0 | 0 | 0 | 0 | 0 | 0 | 0 | 0.16 | 3 |
| unidentified egret/heron* | NA | Pelecaniformes | 0 | 0 | 1.91 | 0 | 0 | 0 | 0 | 0 | 0 | 0 | 0 | 0 | 0 | 0.16 | 3 |
| northern shoveler* | *Spatula clypeata* | Anseriformes | 0 | 0 | 0 | 0.81 | 0.58 | 0 | 0 | 0 | 0 | 0 | 0 | 0 | 0 | 0.14 | 2 |
| unidentified large bird | NA | Unidentified | 0 | 0 | 0 | 0 | 0.3 | 0 | 0 | 0 | 0 | 0 | 2.42 | 0 | 0 | 0.13 | 2 |
| pied-billed grebe^ | *Podilymbus podiceps* | Podicipediformes | 0 | 0 | 0 | 0.65 | 0 | 0 | 0 | 0 | 0 | 0 | 0 | 0.25 | 0 | 0.13 | 2 |
| black-headed grosbeak | *Pheucticus melanocephalus* | Passeriformes | 0 | 0 | 0 | 0 | 0.84 | 0 | 0 | 0 | 0 | 0 | 0 | 0 | 0 | 0.12 | 1 |
| black-crowned night-heron* | *Nycticorax nycticorax* | Pelecaniformes | 0 | 0 | 1.27 | 0 | 0 | 0 | 0 | 0 | 0 | 0 | 0 | 0 | 0 | 0.11 | 2 |
| unidentified hummingbird | NA | Apodiformes | 0 | 0 | 0 | 1.46 | 0 | 0 | 0 | 0 | 0 | 0 | 0 | 0 | 0 | 0.1 | 1 |
| house wren | *Troglodytes aedon* | Passeriformes | 0 | 0 | 0 | 1.39 | 0 | 0 | 0 | 0 | 0 | 0 | 0 | 0 | 0 | 0.09 | 1 |
| black-throated gray warbler | *Setophaga nigrescens* | Passeriformes | 0 | 0 | 0 | 1.29 | 0 | 0 | 0 | 0 | 0 | 0 | 0 | 0 | 0 | 0.09 | 1 |
| least sandpiper* | *Calidris minutilla* | Charadriiformes | 0 | 0 | 0 | 1.29 | 0 | 0 | 0 | 0 | 0 | 0 | 0 | 0 | 0 | 0.09 | 1 |
| Wilson's warbler | *Cardellina pusilla* | Passeriformes | 0 | 0 | 0 | 1.29 | 0 | 0 | 0 | 0 | 0 | 0 | 0 | 0 | 0 | 0.09 | 1 |
| yellow warbler | *Setophaga petechia* | Passeriformes | 0 | 0 | 0 | 1.29 | 0 | 0 | 0 | 0 | 0 | 0 | 0 | 0 | 0 | 0.09 | 1 |
| ring-necked pheasant | *Phasianus colchicus* | Galliformes | 0 | 0 | 0 | 1.05 | 0 | 0 | 0 | 0 | 0 | 0 | 0 | 0 | 0 | 0.07 | 2 |
| osprey* | *Pandion haliaetus* | Accipitriformes | 0 | 0 | 0 | 0 | 0 | 0 | 0 | 0.53 | 0 | 0 | 0 | 0 | 0 | 0.07 | 1 |
| unidentified goose* | NA | Anseriformes | 0 | 0 | 0 | 0 | 0 | 0 | 0 | 0.53 | 0 | 0 | 0 | 0 | 0 | 0.07 | 1 |
| great horned owl | *Bubo virginianus* | Strigiformes | 0 | 0 | 0 | 0 | 0 | 4.12 | 0 | 0 | 0 | 0 | 0 | 0 | 0 | 0.07 | 1 |
| blue-winged teal* | *Spatula discors* | Anseriformes | 0 | 0 | 0 | 0.81 | 0 | 0 | 0 | 0 | 0 | 0 | 0 | 0 | 0 | 0.05 | 1 |
| cinnamon teal* | *Anas cyanoptera* | Anseriformes | 0 | 0 | 0 | 0.81 | 0 | 0 | 0 | 0 | 0 | 0 | 0 | 0 | 0 | 0.05 | 1 |
| Cooper's hawk | *Accipiter cooperii* | Accipitriformes | 0 | 0 | 0 | 0.81 | 0 | 0 | 0 | 0 | 0 | 0 | 0 | 0 | 0 | 0.05 | 1 |
| great-tailed grackle | *Quiscalus mexicanus* | Passeriformes | 0 | 0 | 0 | 0.81 | 0 | 0 | 0 | 0 | 0 | 0 | 0 | 0 | 0 | 0.05 | 1 |
| northern pintail* | *Anas acuta* | Anseriformes | 0 | 0 | 0 | 0.81 | 0 | 0 | 0 | 0 | 0 | 0 | 0 | 0 | 0 | 0.05 | 1 |
| unidentified egret* | NA | Pelecaniformes | 0 | 0 | 0 | 0 | 0.36 | 0 | 0 | 0 | 0 | 0 | 0 | 0 | 0 | 0.05 | 1 |
| brant* | *Branta bernicla* | Anseriformes | 0 | 0 | 0.64 | 0 | 0 | 0 | 0 | 0 | 0 | 0 | 0 | 0 | 0 | 0.05 | 1 |
| common gallinule* | *Gallinula galeata* | Gruiformes | 0 | 0 | 0.64 | 0 | 0 | 0 | 0 | 0 | 0 | 0 | 0 | 0 | 0 | 0.05 | 1 |
| Neotropic cormorant^ | *Phalacrocorax brasilianus* | Suliformes | 0 | 0 | 0.64 | 0 | 0 | 0 | 0 | 0 | 0 | 0 | 0 | 0 | 0 | 0.05 | 1 |
| unidentified buteo | *Buteo spp* | Accipitriformes | 0 | 0 | 0.64 | 0 | 0 | 0 | 0 | 0 | 0 | 0 | 0 | 0 | 0 | 0.05 | 1 |
| double-crested cormorant^ | *Phalacrocorax auritus* | Suliformes | 0 | 0 | 0 | 0.53 | 0 | 0 | 0 | 0 | 0 | 0 | 0 | 0 | 0 | 0.04 | 1 |
| *water-associated birds; ^water-obligate birds; Water-associates are species that rely on water for foraging, reproduction, and/or roosting; water-obligates are species that cannot take flight from land. NA = not applicable. | | | | | | | | | | | | | | | | | |
