# Supplementary material for: A summary of bird mortality at photovoltaic utility scale solar facilities in the Southwestern U.S
Source: PLoS One. 2020 Apr 24;15(4):e0232034. doi: 10.1371/journal.pone.0232034 (PMC7182256; doi:10.1371/journal.pone.0232034)
Supplement: S2 Appendix — (DOCX) [file pone.0232034.s002.docx]

**S2 Appendix. Photovoltaic solar facility studies from January 1, 2013 to September 1, 2018 included in the dataset, with acronym and citation.**

| **Facility** | **Site Acronym** | **Study Acronym** | **Citation** |
| --- | --- | --- | --- |
| **California Valley Solar Ranch** | CC1 | CC1-2 | [1] |
| **Topaz** | CC2 | CC2-1 | [2] |
| **Luning Solar Energy Project** | GB1 | GB1-1 | [3] |
| **Blythe** | SMD1 | SMD1-1 | [4] |
| **Blythe** | SMD1 | SMD1-2 | [5] |
| **Centinela** | SMD2 | SMD2-1 | [6] |
| **Desert Sunlight** | SMD3 | SMD3-1 | [7] |
| **Desert Sunlight** | SMD3 | SMD3-2 | [8] |
| **Longboat Solar Energy Project** | SMD4 | SMD4-1 | [9] |
| **McCoy** | SMD5 | SMD5-1 | [10] |
| **McCoy** | SMD5 | SMD5-2 | [11] |
| **Seville Solar Project** | SMD6 | SMD6-1 | [12] |
| **Silver State South** | SMD7 | SMD7-1 | [13] |

1. H.T. Harvey & Associates. California Valley Solar Ranch Project avian and bat protection plan: final postconstruction fatality report. Unpublished report prepared for HPR II, LLC; 2015.

2. Althouse and Meade, Inc. Topaz Solar Farms 2013 fourth quarter/second annual report for avian and bat protection plan and bird monitoring and avoidance plan; 2014.

3. Moqtaderi C, Riser-Espinoza D, Kosciuch K. Post-construction monitoring, Luning Solar Energy Project, Mineral County, Nevada. June 1, 2017 – May 31, 2018 monitoring report; 2018.

4. Western EcoSystems Technology, Inc. Post-construction monitoring at the Blythe Solar Power Project, Riverside County, California. First annual report draft: 2016 – 2017; 2018.

5. Martinson L, Gerringer M, Lombardi, J. Post-construction monitoring at the Blythe Solar Power Project, Riverside County, California. Second annual report draft: 2017 – 2018; 2019.

6. Heritage Environmental Consultants, LLC. Post-construction avian mortality monitoring report, Centinela Solar Energy Project. Fourth quarterly report (May-June-July, 2015) and annual summary report; 2015.

7. Western EcoSystems Technology, Inc. Avian and bat monitoring at the Desert Sunlight Solar Farm Project, Riverside County, California. 2015 – 2016 annual report; 2017.

8. Western EcoSystems Technology, Inc. Avian and bat monitoring at the Desert Sunlight Solar Farm Project, Riverside County, California. Draft 2016 – 2017 annual report; 2018.

9. Moqtaderi D, Riser-Espinoza D, Kosciuch K. Post-construction monitoring and avian point counts, Longboat Solar Energy Project, San Bernardino County, California. September 1, 2017 – September 1, 2018 monitoring report; 2019.

10. Martinson L, Gerringer M, Lombardi J. Post-construction monitoring at the

McCoy Solar Energy Project, Riverside County, California. Draft first annual report:

2016 – 2017; 2018.

11. Martinson L, Gerringer M, Lombardi J. Post-construction monitoring at the McCoy Solar Energy Project, Riverside County, California. Draft year 2 annual report: 2017 – 2018; 2019.

12. Environmental Resources Management. Seville Solar Project Post-construction mortality monitoring year 1 report; 2018.

13. Gerringer M, Moqtaderi C, Riser-Espinoza D, Martinson L. Post-construction mortality monitoring at the Silver State South Solar Power Project, Clark County, Nevada. Draft first annual report: 2016 – 2017; 2018.
