# Supplementary material for: A summary of bird mortality at photovoltaic utility scale solar facilities in the Southwestern U.S
Source: PLoS One. 2020 Apr 24;15(4):e0232034. doi: 10.1371/journal.pone.0232034 (PMC7182256; doi:10.1371/journal.pone.0232034)
Supplement: S3 Appendix — (DOCX) [file pone.0232034.s003.docx]

**S3 Appendix. Physical attributes and study information for photovoltaic solar facility studies included in the dataset.**

| **Facility** | **Acronym Code** | **Year** | **Study Period** | **Location** | **Bird Conservation Region** | **Nameplate Capacity (Megawatts)** | **Technology** |
| --- | --- | --- | --- | --- | --- | --- | --- |
| **California Valley Solar Ranch** | CC1-2 | 2 | November 7, 2013, to November 17, 2014 | San Luis Obispo County, CA | Coastal California | 250 | Tracker |
| **Topaz** | CC2-1 | 1 | January to December, 2013 | San Luis Obispo County, CA | Coastal California | 550 | Fixed |
| **Luning Solar Energy Project** | GB1-1 | 1 | June 1, 2017, to May 31, 2018 | Mineral County, NV | Great Basin | 50 | Tracker |
| **Blythe** | SMD1-1 | 1 | March 1, 2016, to February 28, 2017 | Riverside County, CA | Sonoran and Mojave Deserts | 235 | Tracker |
| **Blythe** | SMD1-2 | 2 | March 1, 2017, to February 28, 2018 | Riverside County, CA | Sonoran and Mojave Deserts | 235 | Tracker |
| **Centinela** | SMD2-1 | 1 | August 2014 to July 2015 | Imperial County, CA | Sonoran and Mojave Deserts | 170 | Tracker |
| **Desert Sunlight** | SMD3-1 | 1 | February 2, 2015, to February 28, 2016 | Riverside County, CA | Sonoran and Mojave Deserts | 550 | Fixed |
| **Desert Sunlight** | SMD3-2 | 2 | February 29, 2016, to February 28, 2017 | Riverside County, CA | Sonoran and Mojave Deserts | 550 | Fixed |
| **Longboat Solar Energy Project** | SMD4-1 | 1 | September 1, 2017, to September 1, 2018 | San Bernardino County, CA | Sonoran and Mojave Deserts | 20 | Tracker |
| **McCoy** | SMD5-1 | 1 | March 1, 2016, to February 26, 2017 | Riverside County, CA | Sonoran and Mojave Deserts | 250 | Tracker |
| **McCoy** | SMD5-2 | 2 | February 27, 2017, to February 26, 2018 | Riverside County, CA | Sonoran and Mojave Deserts | 250 | Tracker |
| **Seville Solar Project** | SMD6-1 | 1 | February 14, 2017, to February 28, 2018 | Imperial County, CA | Sonoran and Mojave Deserts | 50 | Tracker |
| **Silver State South** | SMD7-1 | 1 | September 1, 2016, to August 31, 2017 | Clark County, NV | Sonoran and Mojave Deserts | 250 | Tracker |
