# Supplementary material for: A summary of bird mortality at photovoltaic utility scale solar facilities in the Southwestern U.S
Source: PLoS One. 2020 Apr 24;15(4):e0232034. doi: 10.1371/journal.pone.0232034 (PMC7182256; doi:10.1371/journal.pone.0232034)
Supplement: S4 Appendix — (DOCX) [file pone.0232034.s004.docx]

**S4 Appendix. Study attributes for photovoltaic solar facility studies from January 1, 2013 to September 1, 2018 included in the dataset.**

| **Facility** | **Acronym Code** | **Fatality Estimates** | **Sampling Percentage (%)** | **Search Type/Viewshed^b^** | **Search Interval** |
| --- | --- | --- | --- | --- | --- |
| **California Valley Solar Ranch** | CC1-2 | Yes | 20 | Walking down every other row, visually scanning intervening rows | Weekly year-round, with daily and 5-day repeat surveys at select sample units |
| **Topaz** | CC2-1 | No | 0.67 | Walking down rows | Daily for a 7-day period each month; different arrays each search |
| **Luning Solar Energy Project** | GB1-1 | Yes | 50 | Walking down rows | Weekly in spring and fall, once every 3 weeks in summer and winter |
| **Blythe** | SMD1-1 | Yes | 41 | Distance sampling; 95-m viewshed | Weekly spring and fall migration, 21 days winter and summer |
| **Blythe** | SMD1-2 | Yes | 41 | Distance sampling; 95-m viewshed | Weekly spring and fall migration, 21 days winter and summer |
| **Centinela** | SMD2-1 | Yes^a^ | 10 | Not specified | Daily for a 7-day period each month |
| **Desert Sunlight** | SMD3-1 | Yes | 30 | Distance sampling; up to 70-m viewshed in Year 1 | Weekly spring and fall migration, 21 days winter and summer |
| **Desert Sunlight** | SMD3-2 | Yes | 41.6 | Distance sampling; up to 140-m viewshed in Year 2 | Weekly spring and fall migration, 21 days winter and summer |
| **Longboat Solar Energy Project** | SMD4-1 | Yes | 100 | Walking every third row, as well as each adjacent row | Every 14 days in spring and fall, once per month in summer and winter |
| **McCoy** | SMD5-1 | Yes | 45 | Distance sampling; 160-m viewshed | Weekly spring and fall migration, 21 days winter and summer |
| **McCoy** | SMD5-2 | Yes | 45 | Distance sampling; 160-m viewshed | Weekly spring and fall migration, 21 days winter and summer |
| **Seville Solar Project** | SMD6-1 | Yes | 27 | Walking down a subset of rows, could view adjacent rows | Bi-monthly in spring and fall, monthly in summer and winter |
| **Silver State South** | SMD7-1 | Yes | 44 | Distance sampling; 71-m viewshed for SB; 150-m for LB | Weekly spring and fall migration, 21 days winter and summer |
| ^a^Fatalities for Centinela were estimated, however, estimates are not compatible with other studies  ^b^SB = small birds, LB = large birds, m = meters | | | | | |
