# Supplementary material for: A summary of bird mortality at photovoltaic utility scale solar facilities in the Southwestern U.S
Source: PLoS One. 2020 Apr 24;15(4):e0232034. doi: 10.1371/journal.pone.0232034 (PMC7182256; doi:10.1371/journal.pone.0232034)
Supplement: S5 Appendix — (DOCX) [file pone.0232034.s005.docx]

**S5 Appendix. Detection bias estimates for photovoltaic solar facility studies from January 1, 2013 to September 1, 2018 included in the dataset.**

| **Facility** | **Acronym Code** | **Year** | **Visibility Class** | **Season** | **Average Detection Probability (Searcher Efficiency * Persistence Probability)** | | |
| --- | --- | --- | --- | --- | --- | --- | --- |
|  |  |  |  |  | **Small Bird** | **Medium Bird** | **Large Bird** |
| Desert Sunlight | SMD3-1 | 1 | Easy | Spring | 0.36 | 0.66 | 0.82 |
| Desert Sunlight | SMD3-1 | 1 | Easy | Summer | 0.38 | 0.54 | 0.58 |
| Desert Sunlight | SMD3-1 | 1 | Easy | Fall | 0.41 | 0.53 | 0.82 |
| Desert Sunlight | SMD3-1 | 1 | Easy | Winter | 0.28 | 0.43 | 0.58 |
| Desert Sunlight | SMD3-1 | 1 | Difficult | Spring | 0.23 | 0.66 | 0.82 |
| Desert Sunlight | SMD3-1 | 1 | Difficult | Summer | 0.24 | 0.54 | 0.58 |
| Desert Sunlight | SMD3-1 | 1 | Difficult | Fall | 0.26 | 0.53 | 0.82 |
| Desert Sunlight | SMD3-1 | 1 | Difficult | Winter | 0.18 | 0.43 | 0.58 |
| Desert Sunlight | SMD3-2 | 2 | Easy | Spring | 0.23 | 0.33 | 0.64 |
| Desert Sunlight | SMD3-2 | 2 | Easy | Summer | 0.08 | 0.22 | 0.75 |
| Desert Sunlight | SMD3-2 | 2 | Easy | Fall | 0.14 | 0.25 | 0.53 |
| Desert Sunlight | SMD3-2 | 2 | Easy | Winter | 0.08 | 0.14 | 0.16 |
| Blythe | SMD1-1 | 1 | All | Spring | 0.28 | 0.37 | 0.76 |
| Blythe | SMD1-1 | 1 | All | Summer | 0.16 | 0.34 | 0.8 |
| Blythe | SMD1-1 | 1 | All | Fall | 0.24 | 0.32 | 0.77 |
| Blythe | SMD1-1 | 1 | All | Winter | 0.07 | 0.13 | 0.39 |
| McCoy | SMD5-1 | 1 | All | Spring | 0.19 | 0.28 | 0.61 |
| McCoy | SMD5-1 | 1 | All | Summer | 0.11 | 0.24 | 0.68 |
| McCoy | SMD5-1 | 1 | All | Fall | 0.16 | 0.22 | 0.63 |
| McCoy | SMD5-1 | 1 | All | Winter | 0.05 | 0.11 | 0.19 |
| Silver State South | SMD7-1 | 1 | All | Spring | 0.32 | NA | 0.60 |
| Silver State South | SMD7-1 | 1 | All | Summer | 0.23 | NA | 0.49 |
| Silver State South | SMD7-1 | 1 | All | Fall | 0.23 | NA | 0.49 |
| Silver State South | SMD7-1 | 1 | All | Winter | 0.23 | NA | 0.31 |
| Blythe | SMD1-2 | 2 | Easy | Spring | 0.24 | 0.44 | 0.71 |
| Blythe | SMD1-2 | 2 | Easy | Summer | 0.03 | 0.27 | 0.74 |
| Blythe | SMD1-2 | 2 | Easy | Fall | 0.18 | 0.34 | 0.76 |
| Blythe | SMD1-2 | 2 | Easy | Winter | 0.05 | 0.16 | 0.48 |
| Blythe | SMD1-2 | 2 | Difficult | Spring | 0.10 | 0.20 | 0.36 |
| Blythe | SMD1-2 | 2 | Difficult | Summer | 0.02 | 0.13 | 0.38 |
| Blythe | SMD1-2 | 2 | Difficult | Fall | 0.09 | 0.17 | 0.39 |
| Blythe | SMD1-2 | 2 | Difficult | Winter | 0.03 | 0.08 | 0.24 |
| McCoy | SMD5-2 | 2 | All | Spring | 0.18 | 0.36 | 0.64 |
| McCoy | SMD5-2 | 2 | All | Summer | 0.02 | 0.20 | 0.67 |
| McCoy | SMD5-2 | 2 | All | Fall | 0.12 | 0.25 | 0.69 |
| McCoy | SMD5-2 | 2 | All | Winter | 0.03 | 0.13 | 0.43 |
| Luning | GB1-1 | 1 | All | Spring | 0.18 | NA | 0.32 |
| Luning | GB1-1 | 1 | All | Summer | 0.13 | NA | 0.14 |
| Luning | GB1-1 | 1 | All | Fall | 0.18 | NA | 0.32 |
| Luning | GB1-1 | 1 | All | Winter | 0.03 | NA | 0.14 |
| Longboat | SMD4-1 | 1 | All | Spring | 0.17 | 0.15 | 0.47 |
| Longboat | SMD4-1 | 1 | All | Summer | 0.12 | 0.45 | 0.78 |
| Longboat | SMD4-1 | 1 | All | Fall | 0.17 | 0.93 | 0.87 |
| Longboat | SMD4-1 | 1 | All | Winter | 0.12 | 0.406 | 0.43 |
